# Targets of light signalling in *Trichoderma reesei*

**DOI:** 10.1186/1471-2164-14-657

**Published:** 2013-09-26

**Authors:** Doris Tisch, Monika Schmoll

**Affiliations:** 1 Research Area of Gene Technology and Applied Biochemistry, Institute for Chemical Engineering, Vienna University of Technology, Gumpendorferstraße 1a, Wien A-1060, Austria; 2 Department Health and Environment – Bioresources, AIT Austrian Institute of Technology, Konrad-Lorenz Strasse 24, Tulln 3430, Austria

**Keywords:** *Trichoderma reesei*, *Hypocrea jecorina*, Light response, Glycoside hydrolases, D-galactose, L-arabinose, ENVOY, BLR1, BLR2

## Abstract

**Background:**

The tropical ascomycete *Trichoderma reesei* (*Hypocrea jecorina*) represents one of the most efficient plant cell wall degraders. Regulation of the enzymes required for this process is affected by nutritional signals as well as other environmental signals including light.

**Results:**

Our transcriptome analysis of strains lacking the photoreceptors BLR1 and BLR2 as well as ENV1 revealed a considerable increase in the number of genes showing significantly different transcript levels in light and darkness compared to wild-type. We show that members of all glycoside hydrolase families can be subject to light dependent regulation, hence confirming nutrient utilization including plant cell wall degradation as a major output pathway of light signalling. In contrast to *N. crassa*, photoreceptor mediated regulation of carbon metabolism in *T. reesei* occurs primarily by BLR1 and BLR2 via their positive effect on induction of *env1* transcription, rather than by a presumed negative effect of ENV1 on the function of the BLR complex. Nevertheless, genes consistently regulated by photoreceptors in *N. crassa* and *T. reesei* are significantly enriched in carbon metabolic functions. Hence, different regulatory mechanisms are operative in these two fungi, while the light dependent regulation of plant cell wall degradation appears to be conserved.

Analysis of growth on different carbon sources revealed that the oxidoreductive D-galactose and pentose catabolism is influenced by light and ENV1. Transcriptional regulation of the target enzymes in these pathways is enhanced by light and influenced by ENV1, BLR1 and/or BLR2. Additionally we detected an ENV1-regulated genomic cluster of 9 genes including the D-mannitol dehydrogenase gene *lxr1,* with two genes of this cluster showing consistent regulation in *N. crassa*.

**Conclusions:**

We show that one major output pathway of light signalling in *Trichoderma reesei* is regulation of glycoside hydrolase genes and the degradation of hemicellulose building blocks. Targets of ENV1 and BLR1/BLR2 are for the most part distinct and indicate individual functions for ENV1 and the BLR complex besides their postulated regulatory interrelationship.

## Background

*Trichoderma reesei* (anamorph of *Hypocrea jecorina*) represents a model system for investigation of plant cell wall degrading enzymes [1,2]. Especially with the current efforts to increase the efficiency and production of cellulase mixtures for economically competitive second generation biofuels, research towards plant cell wall degrading enzymes has gained increased attention [3]. Genetic engineering for strain improvement targets numerous pathways in *T. reesei* and mainly aims at increased production of cellulolytic enzymes [4]. Nutrient availability and utilization are crucial determinants for the survival of *T. reesei* in a natural habitat. Glycoside hydrolases are the main enzymes for this task as they have roles in degradation of biomass (including cellulose and hemicellulose), but they also have functions in defence or pathogenesis and in routine cellular functions such as cell wall remodelling [5]. The genome analysis of *T. reesei*[6] revealed a smaller enzyme set for plant cell wall degradation than expected and recent re-annotation of CAZyme (carbon hydrate active enzymes) genes updated the number of glycoside hydrolases to 201 [7].

Nutrient degradation pathways are tightly regulated in order to ensure maximum efficiency with a minimum of resources used. Therefore *T. reesei* integrates various environmental signals, which result in an adjusted response to the current conditions in its surroundings [8]. In recent years, light response emerged as an important reaction to the environment, which is not only applied in the natural habitats, but is still operative under artificial conditions in the lab or in a fermenter [9]. In *T. reesei*, almost 3% of all genes are differentially regulated in light and darkness and these genes are enriched in functions of carbohydrate transport and metabolism [10]. *T. reesei* possesses homologues of the two *N. crassa* photoreceptors White Collar-1 (WC-1) and White Collar-2 (WC-2) - two zinc-finger transcription factors, which usually act as a complex [11,12]. The *T. reesei* homologues BLR1 and BLR2 (blue light regulator 1 and 2) are involved in regulation of cellulase gene expression [13,14] and to some extent in asexual and sexual development [13,15]. The third *N. crassa* photoreceptor, VIVID (VVD) is responsible for gating of light responses [16], acts negatively on the White collar complex (WCC) and can act as a universal brake on light response [17-21]. Its orthologue in *T. reesei*, ENV1, was found to have a profound effect on light dependent processes and signalling, including regulation of cellulase gene expression [13,22-24]. However, despite a number of similar functions of VVD and ENV1, also in cellulase regulation [25], these factors are not functional homologues [22]. In contrast to *N. crassa* strains lacking functional VVD [16], deletion strains of ENV1 show a severe growth defect in light [13,22,24]. Additionally, ENV1 is essential for female fertility of *T. reesei* in light [15], is assumed to influence cAMP turnover by a negative effect on phosphodiesterases and impacts regulation of the heterotrimeric G-protein pathway [24].

Investigation of the influence of BLR1, BLR2 and ENV1 on efficiency of cellulose degradation under fermentation conditions revealed an influence of all three photoreceptors [14]. The efficiency of the secreted enzyme mixture was enhanced in Δ*env1*, while the higher cellulolytic activity in Δ*blr2* media was due to increased secretion capacity. Strains lacking *blr1* did show enhanced biomass accumulation in the presence of cellulose, but production of hydrolytic enzymes was nevertheless weakest in this strain. However, comparison of transcript levels of cellulases with cellulolytic activity in the culture filtrate or abundance of the respective protein there, revealed discrepancies, especially in light [13,14,22,26]. Hence, an influence of components involved in perception and transmission of the light signal could also be responsible for posttranscriptional and/or posttranslational regulation of enzyme production.

Utilization of plant cell walls not only involves the uptake of the building blocks of cellulose, but also components released from hemicellulose, such as galactose or arabinose are encountered on this natural substrate and channelled into catabolic pathways [27,28]. For catabolism of D-galactose, different pathways are used in fungi. In the Leloir pathway conversion of D-galactose in several steps to D-glucose-6-phosphate involves phosphorylation [28,29]. The alternative oxidoreductive D-galactose pathway has the intermediates D-galactose, galactitol, L-xylo-3-hexulose, D-sorbitol and D-fructose [27]. The enzymes involved in catabolism of D-galactose, aldose reductase XYL1, galactitol dehydrogenase LAD1, L-xylo-3-hexulose reductase LXR4 and D-sorbitol dehydrogenase XDH1 also have functions in degradation of L-arabinose, with additional contribution of the L-xylulose reductase LXR3 [30].

In this study, we investigated genome wide transcriptional regulation by BLR1, BLR2 and ENV1 and we aimed to evaluate different regulatory patterns caused by these factors. We show that the recently discovered imbalance in light dependent gene regulation i. e. an increased number of genes regulated by light [10] is even more pronounced in mutants lacking ENV1 and that around 75% of all genes encoding glycoside hydrolases of *T. reesei* are differentially regulated in light and darkness in wild-type or mutant strains. We also found that catabolism of hemicellulase building blocks is subject to regulation by light, which is in part mediated by BLR1, BLR2 and ENV1 with one genomic cluster being regulated by light upon growth on cellulose.

## Results

### Transcriptome analysis of gene regulation by ENV1, BLR1 and BLR2

We studied differential regulation by ENV1, BLR1 and BLR2 by microarray analysis in light and darkness upon growth on microcrystalline cellulose. Downregulation of the transcription of *env1* and the photolyase gene *phr1* in Δ*blr1* and Δ*blr2* in light as well as the regulation pattern of the cellobiohydrolase gene *cbh2/cel6a* in light and darkness and in Δ*env1* are in accordance with earlier studies [13,22,31]. Additionally, qRT-PCR analysis done for previously reported studies with the same experimental setup [10] and data on evaluation of the genes encoding enzymes involved in degradation of pentoses (this study, see below) was in agreement with microarray results and and hence confirm the validity of our results.

**Figure 1:**
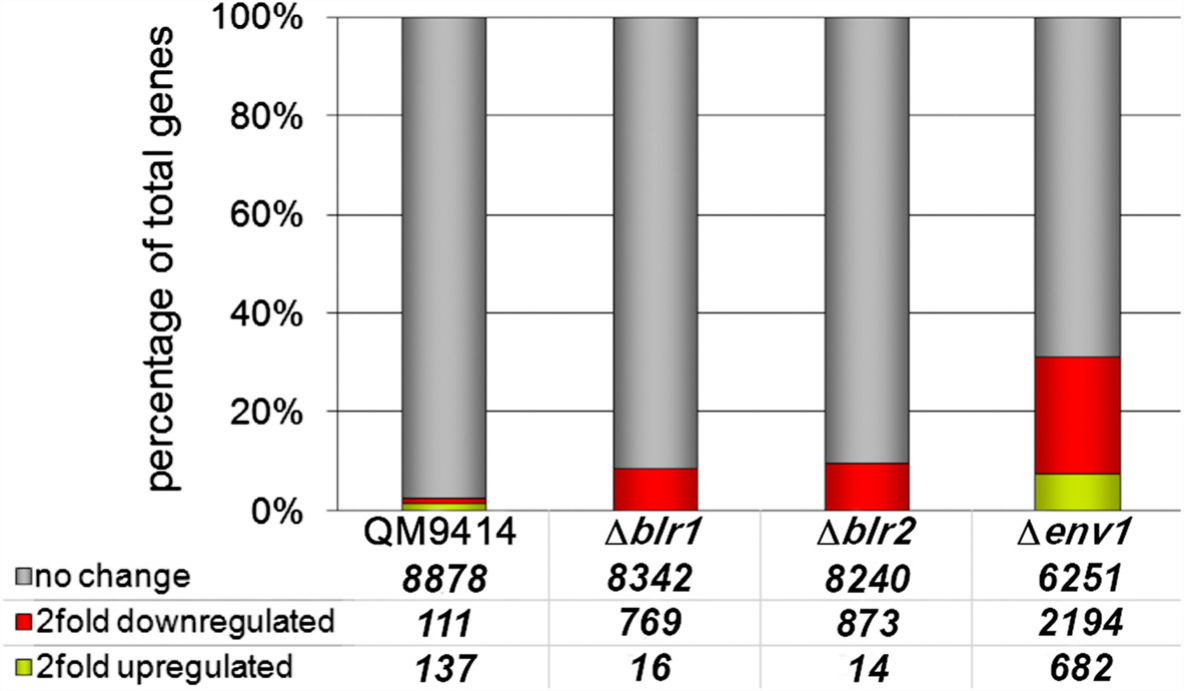
**Comparison of light dependent differential regulation in wild-type, ∆*env1*, ∆*blr1 *and ∆*blr2*.** Genes upregulated in light are represented by a green bar, those down-regulated are shown as red bar.

In order to gain insight into the impact of components of the light signalling machinery on light responsiveness (here meant to describe differential transcription between cultivation in constant light or constant darkness) of transcript abundance, we compared differential gene regulation between light and darkness in Δ*env1*, Δ*blr1* and Δ*blr2*. In the wildtype 2.8% of all genes are regulated in response to light, but this percentage strongly increases upon deletion of the phosducin like protein encoding *phlp1*, *gnb1* or *gng1* (genes encoding the G-protein beta and gamma subunits) up to 23% [10]. Intriguingly, deletion of *blr1* or *blr2* also causes the number of light-dependently regulated genes to increase (up to 9.7% of total genes), which indicates that strains lacking these photoreceptors are defective in proper regulation of light responsiveness or adaptation to constant light, but are not blind (Figure 1). This finding suggests that photoreceptors do not exclusively act as a complex as was already suggested earlier [25] and that additional factors are present in the genome which transmit light signals in the absence of the photoreceptors BLR1, BLR2 and ENV1 as also shown for their orthologues in *N. crassa*[18]. In Δ*env1* the number of light responsive genes strongly increases to 31.6% of all genes of *T. reesei* (2888 genes) (Figure 1). In all three strains, differential transcription between light and darkness was both positive and negative, which is in agreement with data from *T. atroviride*[32], but in contrast to *N. crassa* for which only positive regulation by photoreceptors was observed [18]. Additionally, we found a remarkably high number of glycoside hydrolase encoding genes and signal transduction components among those downregulated due to illumination, but also numerous transcription factors – particularly in Δ*env1*. (Additional file 1). Interestingly, the number of light responsive genes is higher in the Δ*env1* mutant compared to the individual Δ*blr* strains and also higher than both Δ*blr* strains together. This finding confirms earlier results on individual functions of ENV1 [23] and also functions independent of BLR1 and BLR2.

### Functions of genes with differential regulation in Δ*blr1*, Δ*blr2* and Δ*env1*

Due to the considerably increased number of genes differentially regulated between light and darkness in *Δblr1, Δblr2* and *Δenv1*, we were interested, which functions were targeted by this regulation.

In Δ*blr1* only 16 genes were upregulated by light and most of them only around 2fold, among them a putative sulphate permease (TR_79741) and two putative ß-glucosidases (TR_47268 and TR_124175). Also in Δ*blr2* only 14 genes are upregulated in light, including an NMT1-like gene (TR_121620) putatively involved in thiamine biosynthesis and the PTH11 type G-protein coupled receptor gene TR_69500. Only TR_108143 encoding an unknown hypothetical protein is upregulated in Δ*blr1* and Δ*blr2* in light compared to darkness*.*

**Figure 2:**
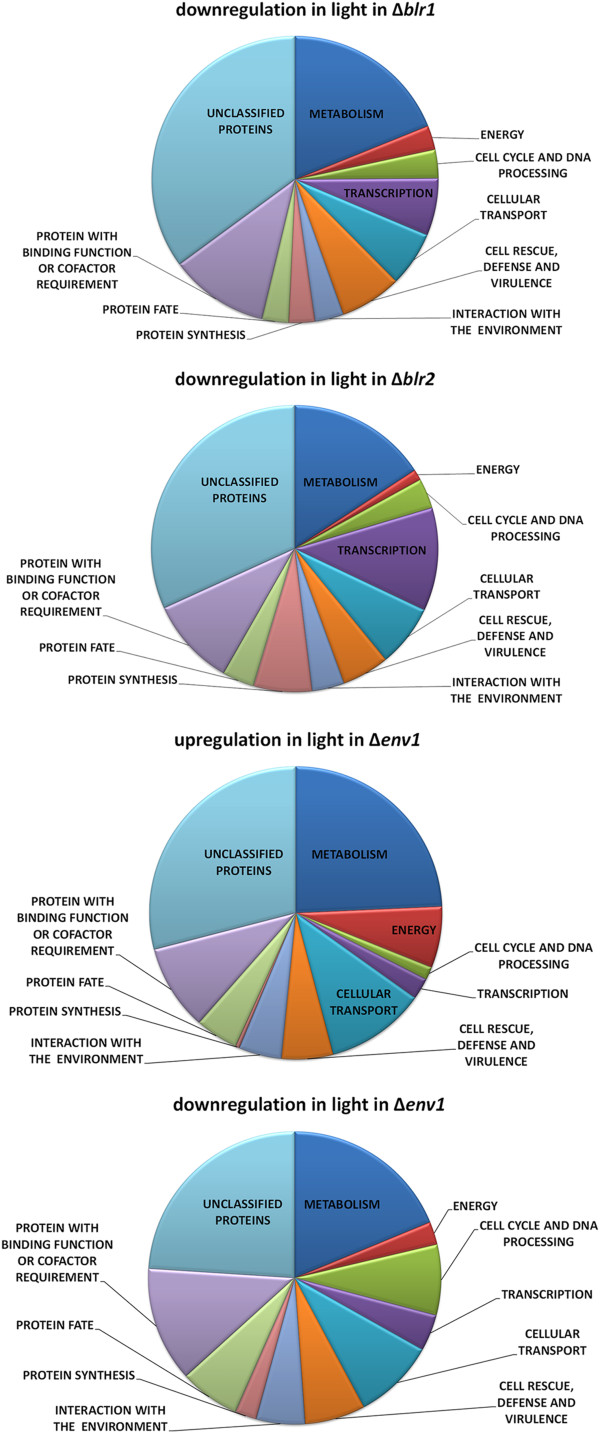
**Functions of genes differentially regulated in light and darkness in ∆** ***env1*, ∆** ***blr1 *** **and ∆** ***blr2.***

In both photoreceptor mutants the majority of genes was downregulated in light (769 in Δ*blr1* and 873 in Δ*blr2*) and exert diverse functions (Figure 2). Detailed analysis revealed that genes significantly enriched in the geneset down regulated in Δ*blr1* in light include metabolism (p-value 2.27 e-05), particularly C-compound and carbohydrate metabolism (p-value 5.90 e-04), secondary metabolism (p-value 5.04 e-04), ABC-transporters (p-value 1.22 e-04), oxygen and radical detoxification (p-value 1.81 e-05) including catalase reaction and superoxide metabolism.

Genes down regulated in Δ*blr2* in light are significantly enriched for functions in transcription (p-value 3.02 e-08) and protein synthesis (p-value 4.82 e-06), but although numerous metabolic genes are regulated (Figure 2), no significant enrichment in this function was observed. These obviously different functions of BLR1 and BLR2 are in agreement with earlier data in *T. atroviride* and *N. crassa*, which indicated independent roles of the two photoreceptors besides their function as complexes [25,33].

For genes upregulated in light in Δ*env1* we observed a significant enrichment in metabolic functions (p-value 3.87 e-21), especially in amino acid metabolism (p-value 5.72 e-11), C-compound and carbohydrate metabolism (p-value 4.6 e-07) and lipid, fatty acid and isoprenoid metabolism (p-value 9.85 e-17). Moreover, genes with functions in energy supply, oxidation of fatty acids and cellular transport, particularly C-compound and carbohydrate transport were significantly enriched. With genes downregulated in Δ*env1* again metabolic functions are significantly enriched (p-value 9.92 e-23), with strongest enrichment in C-compound and carbohydrate metabolism (p-value 7.79 e-17), but although metabolism of some amino acids is still enriched, the strong enrichment in amino acid metabolism in general as seen for upregulated genes is not obvious in this gene set. Further enrichment occurred with genes involved in sulphur metabolism and sulphate assimilation (p-value 7.07 e-08), cell cycle and DNA processing (p-value 1.07 e-13). From the latter functional group, also genes involved in DNA recombination and repair, mitotic cell cycle and cell cycle control, cell division and septum formation/hydrolysis are significantly enriched among those downregulated in Δ*env1,* which correlates with its strong growth and developmental defect in light [13,22]. Interestingly, also genes involved in translation are significantly enriched among the downregulated genes (p-value 1.43 e-03), which hints at a function of ENV1 in modulation of posttranscriptional regulation of gene expression as suggested for several genes in light in *T. reesei*[14,26]. Additionally also cellular transport, including C-compound and carbohydrate transport, defence mechanisms, stress response, DNA damage response, cellular sensing and response, development are enriched in this gene set.

In order to put these results into context, we searched in a hierarchical cluster analysis for genes upregulated in light in the wild-type but not in the mutant strains investigated in this study. We found a cluster of 187 genes which fulfilled this criterion. Genes in this cluster were enriched in functions in metabolism (p-value 1.37 e-06) including nitrogen and sugar metabolism and secondary metabolism, which are also among the functions most elaborately regulated in all three mutants.

### The influence of light on transcription of glycoside hydrolase encoding genes is in part mediated by ENV1, BLR1 and BLR2

**Figure 3:**
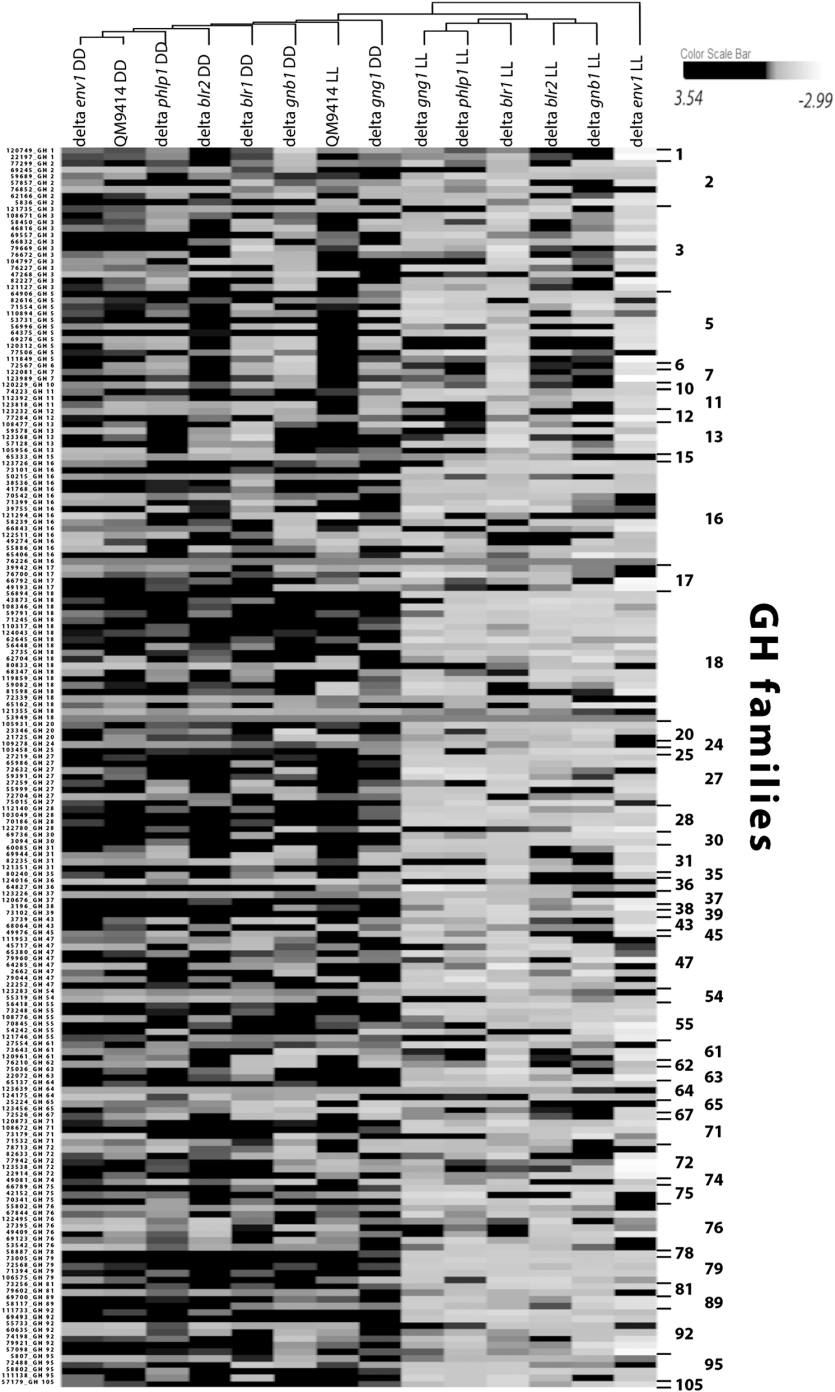
**Overview of light dependent modulation of glycoside hydrolase transcript levels.** Hierarchical clustering analysis of transcript patterns of the wildtype QM9414 and ∆*blr1*, ∆*blr2*, ∆*env1* was performed for constant light and darkness. Data on ∆*gnb1*, ∆*gng1* and ∆*phlp1*[10] were added for comparison and reflect considerable light dependent regulation.

Previously we showed that in signalling mutant strains of the heterotrimeric G-protein pathway, the number of genes differentially regulated in light and darkness increases and that this effect also concerns glycoside hydrolases [10]. Here, we found that deletion of *env1*, *blr1* or *blr2* led to light dependent regulation of 129 glycoside hydrolase genes (Additional file 2: Table S1; Figure 3), partially overlapping with GH encoding genes already known to be potentially light regulated [10]. Together with those genes, which were shown to be regulated by light in mutants in the heterotrimeric G-protein pathway, in total 148 out of 201 genes (75% of total GH encoding genes) were found to be differentially regulated in light and darkness in the wildtype and/or in mutant strains. Among these 148 genes, all GH families are represented except GH family 35, with its only member beta galactosidase (TR_80240). However, inspection of transcript levels of the respective gene also differed between light and darkness up to 1.96fold, which is only slightly below our threshold.

### Functions of ENV1 in darkness

Previous studies showed that ENV1 not only has functions in light, but also in darkness [23], which however remained elusive until now. The transcriptome data confirmed this assumption and 35 genes were found to be specifically regulated more than 2fold (p-value 0.01) in darkness in Δ*env1* (Additional file 3). Only 6 genes were found to be exclusively downregulated in Δ*env1* in darkness, including two putative FAD dependent oxidoreductases (TR_22915 and TR_111357), phosphoenolpyruvate carboxykinase TR_124115 and one predicted oligopeptide transporter (TR_44278).

Those genes showing increased transcript abundance specifically in Δ*env1* in darkness include 3 glycoside hydrolases (TR_5836, TR_111849 (*xyn4*) and TR_70845), the class II hydrophobin encoding gene *hfb3*, one candidate non-ribosomal peptide synthase gene (TR_123786), one catalase (TR_73818), *blr1* and *blr2*. Moreover, three genes involved in sulphur metabolism (TR_103012 and TR_112567, two predicted taurine dioxygenases, and TR_69696) are upregulated. In summary, the functions influenced by ENV1 in darkness correspond to a subset of functions subject to light response in *T. reesei*. These results further reflect that ENV1 exerts individual functions not only in light, but also in darkness.

### Regulatory targets of BLR1 and BLR2 in darkness

In addition to differential regulation between growth in light and growth in darkness, we also studied altered gene regulation in the photoreceptor mutant strains compared to wild-type in light and darkness, which revealed light-dependent regulatory targets of these factors (Additional files 4, 5 and 6).

Previous studies in *T. reesei* indicated that BLR1 and BLR2 not only have functions in light, but that they also regulate gene expression and metabolic functions in darkness [13,14]. q-RT PCR analysis of the cellobiohydrolase *cbh1/cel7a,* for which the microarray reached the saturation limit, confirmed a regulatory function of BLR1 and BLR2 in darkness (Additional file 2: Figure S1). Moreover, individual functions for the two photoreceptors homologues besides their activity as a complex were suggested in *N. crassa*[25]. We therefore analyzed which functions these photoreceptors target in darkness. We found that in Δ*blr1* 128 genes are upregulated in darkness, among them 6 glycoside hydrolases including two alpha-glycosidases (TR_60635 and TR_27395), two beta-glycosidases (TR_55886 and TR_124175), one alpha galactosidase (TR_72704) and one chitinase (TR_80833). Moreover, seven genes involved in sulphur metabolism and 5 transporters are among the genes upregulated in Δ*blr1*. 73 genes were downregulated in Δ*blr1* in darkness. Accordingly, functional category analysis identified C-compound and carbohydrate metabolism in the gene set upregulated in Δ*blr1* and metabolism of several amino acids among the genes down regulated in Δ*blr1* in darkness as significantly enriched (p-values < 0.01). Genes exerting transport functions were significantly enriched in both gene sets in Δ*blr1*.

In Δ*blr2*, 42 genes are upregulated in darkness including 7 glycoside hydrolases, among them two beta glucosidases (TR_46816 and TR_76672) and one L-arabinofuranosidase (TR_76120) and three transporters. Among the genes down-regulated in Δ*blr2*, 7 genes involved in sulphur metabolism were found. Funcat analysis revealed significant enrichment in genes involved in polysaccharide and carbohydrate metabolism as well as transport functions among the genes up- or down-regulated in Δ*blr2* in darkness.

Hence both photoreceptors play a role in alteration of carbohydrate metabolic functions and transport of compounds with distinct, both positive and negative targets in darkness.

### Shared regulatory targets of BLR1, BLR2 and ENV1

**Figure 4:**
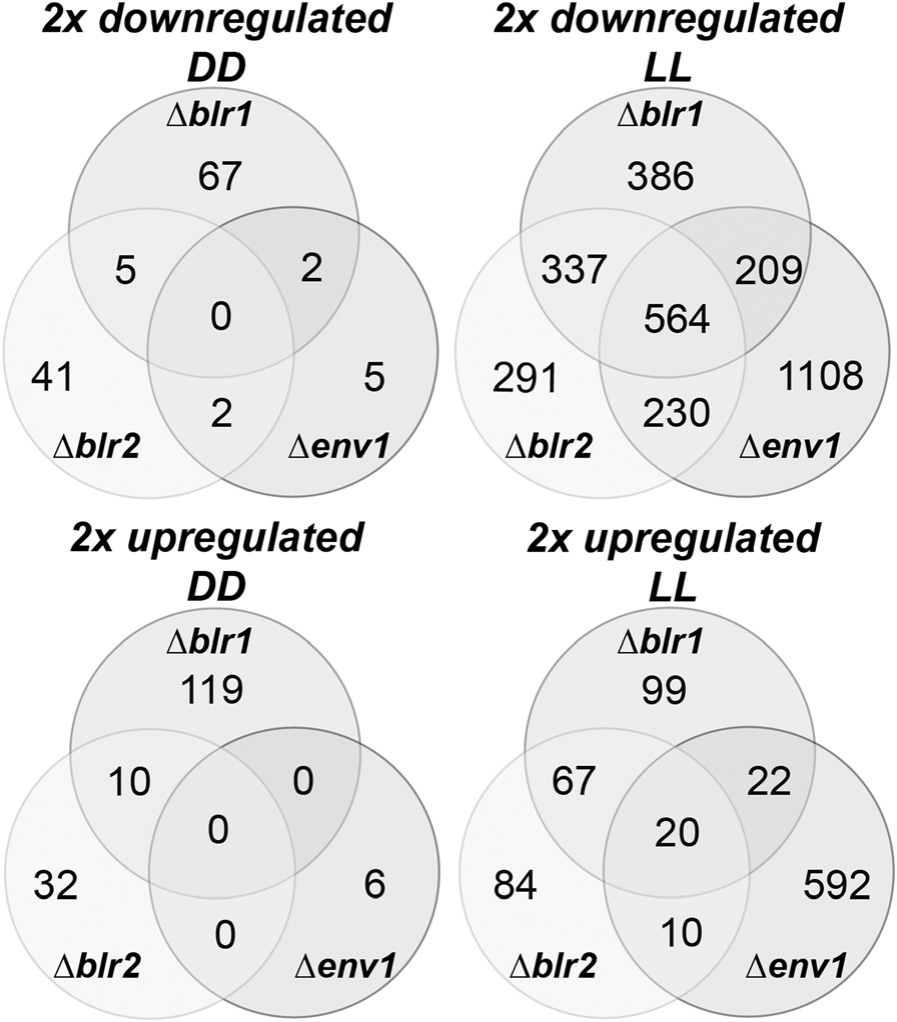
**Overlap of target genes of ENV1, BLR1 and BLR2.** The Venn-diagram shows twofold significantly regulated genes in the deletion strains ∆*env1*, ∆*blr1* and ∆*blr2* in comparison with the parental strain QM9414 in light and darkness.

Light dependent induction of *env1* transcription requires the presence of BLR1 and BLR2 [13]. In order to elucidate which target genes would be regulated by the complex assumed to be formed by BLR1 and BLR2 and transmitted involving the function of ENV1, we screened for genes regulated similarly in Δ*env1*, Δ*blr1* and Δ*blr2* (Figure 4; Additional file 7). While in darkness no overlap could be detected, we found 20 genes to be upregulated in all three mutants in light, which are concluded to represent genes negatively influenced by the BLR1/BLR2 complex via ENV1 in light. Among them were 2 glycoside hydrolase family 16 genes (TR_121294 and TR_49274) as well as one putative zinc binuclear cluster transcription factor (TR_122523). However, in contrast to the only 20 genes negatively influenced by BLR1, BLR2 and ENV1, we found the majority of common targets of these factors (564 genes, Figure 4) to be positively regulated in light by the light signalling machinery. Although most of the genes in this group are of unknown function, major targets appear to be the glycoside hydrolases with 22 members of diverse families found in this group. Additionally, three putative transcription factors (TR_107974, TR_110901 and TR_120365) and two G protein coupled receptors, (TR_57101 and TR_63981) were downregulated in all three mutants in light, suggesting altered signal perception as well as output on regulatory targets. The finding of six genes involved in sulphur metabolism (TR_103012, TR_104081, TR_3823, TR_59876, TR_7625, TR_77795) including the E3 ubiquitin ligase LIM1 [34] supports the hypothesis of a role of this process in light-dependent modulation of gene expression in *T. reesei*. Additionally, the finding of one hydrophobin gene in this group (TR_105869) is also not without precedent [35].

### Regulation by a potential negative effect of ENV1 on the BLR complex

For *N. crassa*, 417 genes were found to be up-regulated in Δ*vvd* and down-regulated in the white-collar mutants upon growth on cellulose in light, which were significantly enriched in C-compound and carbohydrate metabolism. Hence, carbon metabolism was suggested to be subject to photoadaptation in *N. crassa*[25]. In order to evaluate this hypothesis for *T. reesei*, we also screened for genes down-regulated due to the lack of a functional photoreceptor complex (genes downregulated in Δ*blr1* and Δ*blr2*), but upregulated in Δ*env1.* Lack of ENV1 is in this case assumed to result in increased transcription of genes positively regulated by BLR1 and BLR2. We only found 72 genes of those downregulated in Δ*blr1* and Δ*blr2* in light (Figure 4; 337 in total) to be up-regulated in Δ*env1*. Interestingly, these included 5 genes involved in pheromone processing and sexual development, two photolyases as well as two transcription factors (Additional file 8). However, no enrichment in carbon metabolic functions as seen for *N. crassa* was observed. Consequently, in contrast to *N. crassa*, carbon metabolism appears to be regulated by the influence of BLR1 and BLR2 on induction of *env1* rather than the assumed negative effect of ENV1 on the function of the BLR complex in light. Additionally, this discrepancy may reflect the regulatory differences of ENV1 and VVD, which are no functional homologues [22].

### Distinct targets of the BLR1/BLR2 complex versus ENV1

Despite considerable overlap in their major targets in light with ENV1, the common targets of BLR1 and BLR2 (indicating their acting as a complex) are distinct from those of ENV1 to a certain extent (Figures 2 and 4; Additional file 8). 67 genes were found to be upregulated in light compared to the wildtype in both Δ*blr1* and Δ*blr2* but not in Δ*env1*, including one transcription factor (TR_105520), 3 genes involved in sulphur metabolism (TR_22453, TR_62285, TR_79933), one PTH11-type GPCR (TR_69500), one polyketide synthase (TR_73618) and one glycoside hydrolase family 16 gene (TR_122511). 337 genes represent positive targets of the BLR1/BLR2-complex in light, among them were one polyketide synthase from PKS orthologous group 3 [36] (TR_105804) and one non-ribosomal peptide synthase (TR_69946) as well as 4 glycoside hydrolases. Interestingly, we also detected 8 transcription factors positively regulated by BLR1 and BLR2 but not by ENV1 including one transcription factor (TR_57735) reported to be responsive to light in the absence of major photoreceptors [18].

### ENV1 triggers gene regulation also independently of BLR1/BLR2

The results described above led to the question, which output pathways would be regulated by ENV1 independently of BLR1/BLR2 in light. More than 55% (1108 genes) of all genes downregulated and even 92% (592 genes) of those upregulated in a mutant lacking ENV1 are not targets of either BLR1 or BLR2 (Figure 4; Additional file 9). Moreover, the negative effect of ENV1 in light is much more widespread than that of BLR1 or BLR2. Consequently, a function for ENV1 distinct of that of BLR1 and BLR2 in light can be assumed.

Analysis of negative targets of ENV1 in light revealed an influence on 13 putative transcription factors, including two transcription factors (TR_103230, TR_72057) described to be light responsive in the absence of the major photoreceptors in *N. crassa*[18]. Moreover, 3 photolyases (*phr1*, TR_59726, TR_77473), 5 G protein coupled receptors (TR_103694, TR_119819, TR_55561, TR56426, TR_72627), 5 genes involved in secretion (TR_53254, TR_55774, TR_105763, TR_122870, TR_123922), 3 genes involved in secondary metabolism (TR_68204, TR_58285, TR_106272) and 14 glycoside hydrolase genes were negatively regulated by ENV1. Genes influenced positively by ENV1, but not by BLR1 or BLR2 in light include 17 putative transcription factors, among them *hap3*, encoding an important regulator of cellulase gene expression [37]. Additionally, 13 genes involved in sulphur metabolism, eight G protein coupled receptors (five of them belonging to the PTH11-type), nine genes involved in secretion, two polyketide synthases (TR_59482 – PKS orthologous group 5, TR_73621), three hydrophobin genes (TR_73173, TR_119989, TR_123967) and most intriguingly, nine glycoside transferase and 36 glycoside hydrolase genes are enhanced by ENV1 in light. We conclude that ENV1 represents a key factor in light-dependent regulation of gene expression, the major effect of which is not exerted concertedly with the BLR1/BLR2 complex. The considerable number of target genes with functions in carbon utilization and nutrient signal perception suggests a crucial function in interconnecting nutrient with light signalling.

### Coregulation of genes with the major cellulases

Genes regulated consistently under different conditions often participate in the same function. Therefore, investigation of co-regulated genes can help to assign a putative function to unknown genes or reveal processes related to each other [38-40]. For identification of genes and/or processes involved in the light signalling pathway that modulates the cellulase gene transcription pattern, we examined a cluster of 52 genes that are co-regulated with *cel6a/cbh2* in all of the light affected mutant strains and their parental strain QM9414. *cel7a/cbh1*, the major cellobiohydrolase, is co-regulated with *cel6a/cbh2*, but due to saturation of the microarray signal for this transcript, we were not able to use the transcript pattern of *cel7a/cbh1* to evaluate coregulated genes of cellulases. To confirm that the transcription pattern of *cel6a/cbh2* in the mutants resembles that of *cel7a/cbh1*, we performed qRT-PCR, which confirmed coregulation (Additional file 2: Figure S1). We consequently used *cel6a/cbh2* as representative gene for evaluation of genes coregulated with cellobiohydrolases in the presence or absence of BLR1, BLR2 or ENV1 as described above in light and darkness (Additional file 2: Table S2).

The gene set of the *cel6a/cbh2* cluster comprised 12 more glycoside hydrolase family genes, a swollenin (TR_123992), which was shown to disrupt the structure of cellulosic materials [41], two carbohydrate esterase family genes (*axe1*- TR_73632, TR_54219), *cip1* and *cip2 -* each containing a carbohydrate-binding module and were previously shown to be co-expressed with cellulases and represent “novel” types of cellulases [42,43], a flavohemoglobin (TR_76722), a GABA permease (TR_70098) and two potential transcription factors (TR_77154, TR_73654). In addition, two hypothetical proteins, a WD40-repeat containing protein (TR_103064) and a hypothetical G protein coupled receptor (TR_53238) were also coregulated with *cel6a/cbh2*. Notably, the xylanase regulator 1 encoding gene *xyr1* was also shown to be coregulated with *cel7a/cbh1* and *cel6a/cbh2*, which is in agreement with earlier data, because XYR1 influences cellulase gene transcription positively and correlates with cellulase transcription levels [44,45]. However, positive regulation of *xyr1* by light has not been observed upon growth on lactose [46] and appears to be specific to cellulose, which also suggests differential light dependent regulation of plant cell wall degrading enzymes on these carbon sources. Indeed, previous studies of *T. reesei* grown on cellulose or lactose showed that the effect of light on cellulase gene expression is positive on cellulose [22] and negative on lactose [46].

### Light impacts pentose and D-galactose metabolism

**Figure 5:**
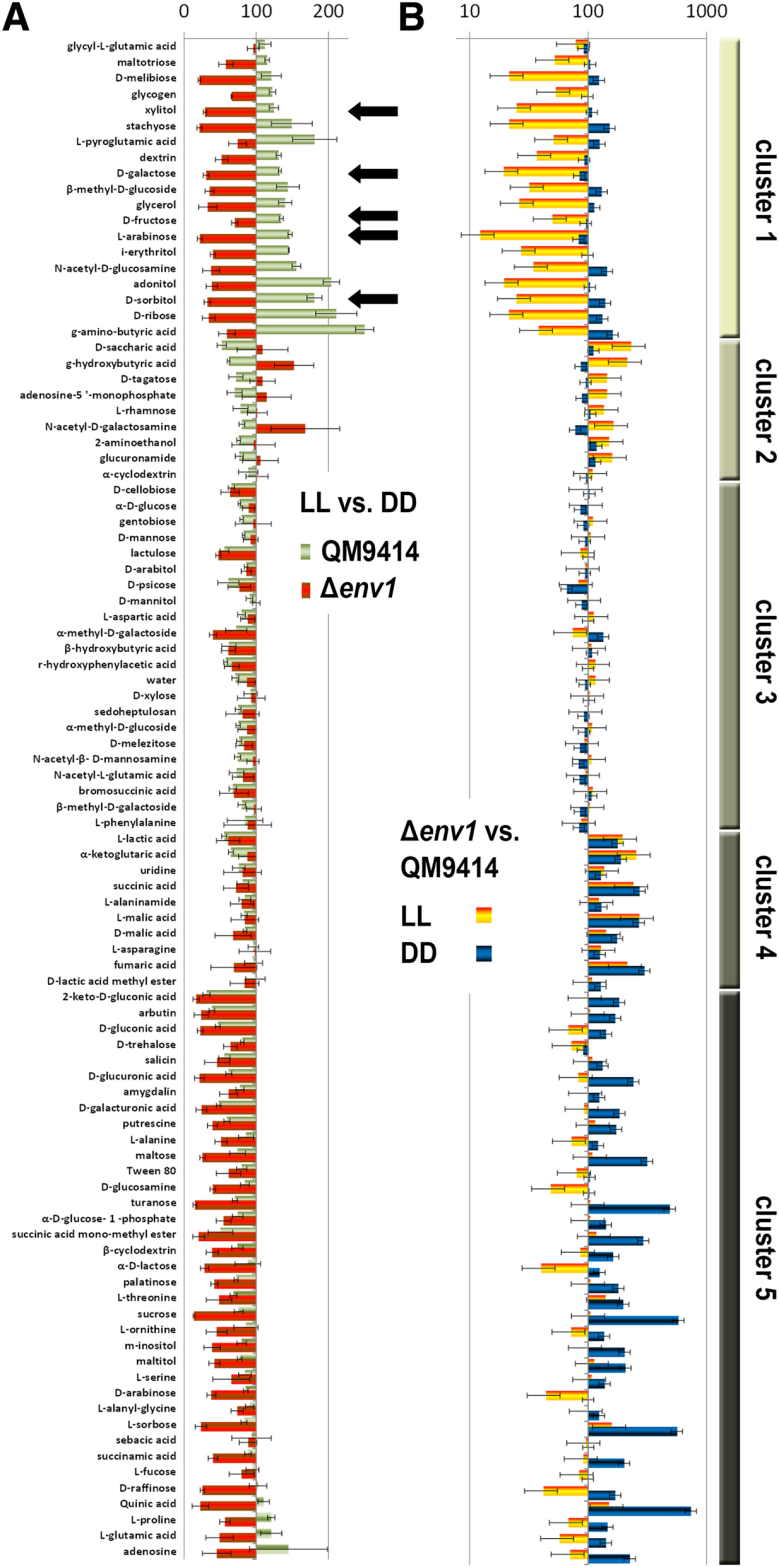
**BIOLOG analysis of growth of QM9414 and env1PAS- in light and darkness.** Strains were grown for 72 hours on 96 different carbon sources [23]. Hierarchical cluster analysis was performed using HCE 3.5 with default settings. Standard deviations shown result from three biological replicates. Values are represented as percent. LL means constant light, DD means constant darkness.

The considerable regulation of glycoside hydrolases and hence of mechanisms involved in substrate degradation by ENV1 led us to re-evaluate data on growth patterns for various carbon sources in more detail [23]. The results for growth on 96 carbon sources in constant light and constant darkness of the parental strain and the ENV1-non functional strain *env1*^PAS–^[13,22] were rearranged and used for hierarchical cluster analysis. *env1*^PAS–^ showed considerably weaker growth in light than in darkness on most carbon sources, as could be expected from its published growth defect in light [13,22] (Figure 5). The growth of the wildtype was enhanced in light in a cluster of 19 different carbon sources including D-sorbitol, L-arabinose, D-fructose, D-galactose and xylitol in light, while the *env1* mutant strain showed a considerably lower growth rate in light on these carbon sources (indicated by an arrow in Figure 5). Hence, the positive influence of light on growth on these carbon sources is likely to be at least in part mediated by ENV1.

### D-galactose and pentose catabolism are regulated by light on cellulose

**Figure 6:**
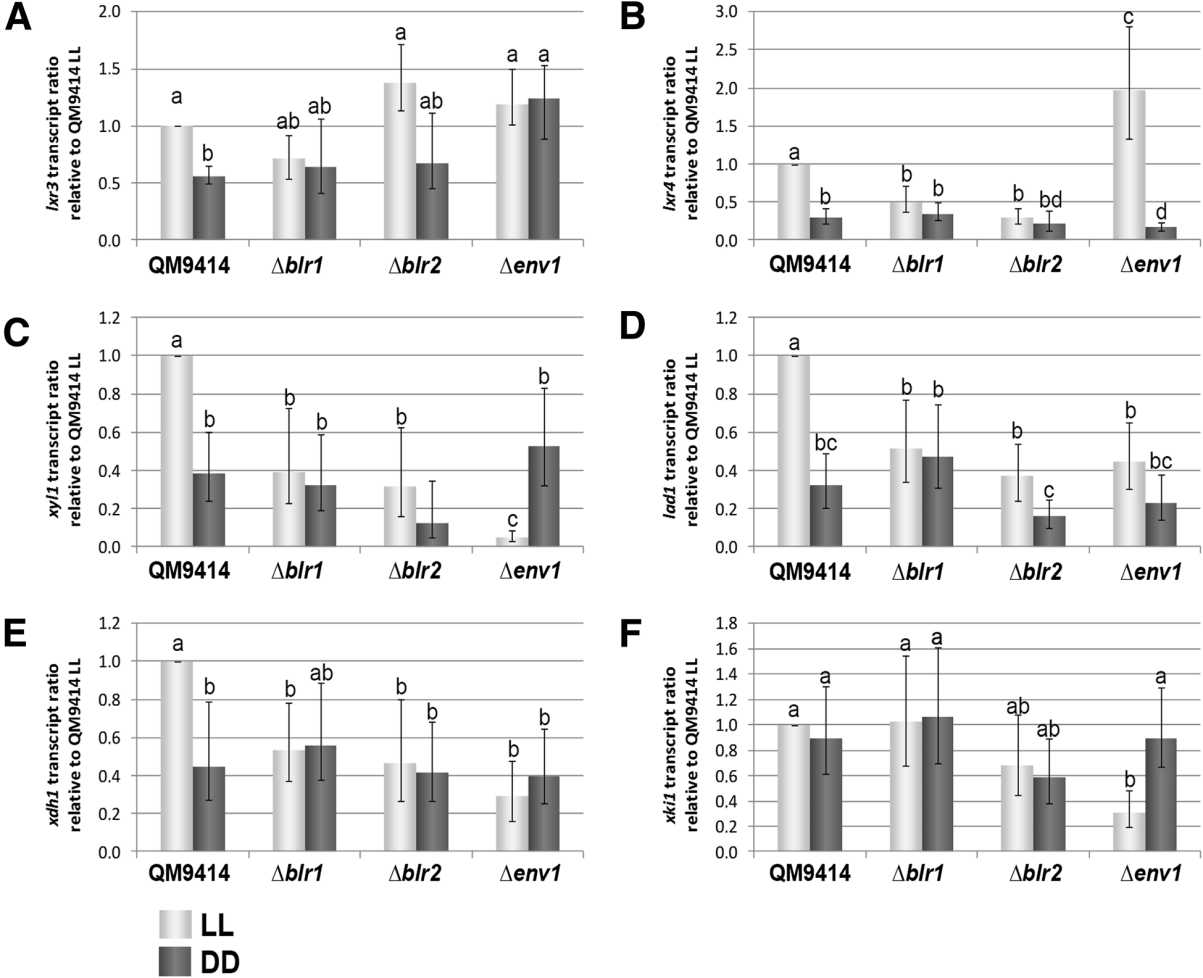
**qRT PCR analysis of transcript abundance of genes involved in D-galactose and L-arabinose metabolism.** Transcript levels of *lxr3***(A)***, lxr4 ***(B)**, *xyl1 ***(C)**, *lad1 ***(D)**, *xdh1 ***(E)** and *xki1 ***(F)** were analyzed in constant light (LL) and constant darkness (DD) on cellulose in QM9414, ∆*env1*, ∆*blr1* and ∆*blr2*. Data from two biological and three technical replicates were included in the calculations. Different letters above the data point reflect statistically significant differential regulation (p-value 0.01; as calculated using the REST software).

Interestingly, this cluster of carbon sources with ENV1 dependent enhanced regulation by light comprises several carbon sources of the D-galactose and pentose catabolism (D-galactose, L-arabinose, D-sorbitol, xylitol and D-fructose). The respective enzymes are assumed to be involved in metabolism of hemicellulose degradation products [47]. Considering coregulation of many plant cell wall degrading enzymes upon detection of an inducing substrate [48], a regulation of these pathways also on cellulose seemed reasonable and was indeed observed with the microarray data of this study, albeit very low expression levels for *lxr3*, *lxr4* and *lad1* as well as only small differences in transcript levels necessitated independent confirmation of these data. Consequently, we evaluated the microarray data by qRT-PCR for the key components of oxidoreductive pentose and D-galactose pathway with respect to transcriptional regulation by light and/or the photoreceptors (Figure 6).

**Figure 7:**
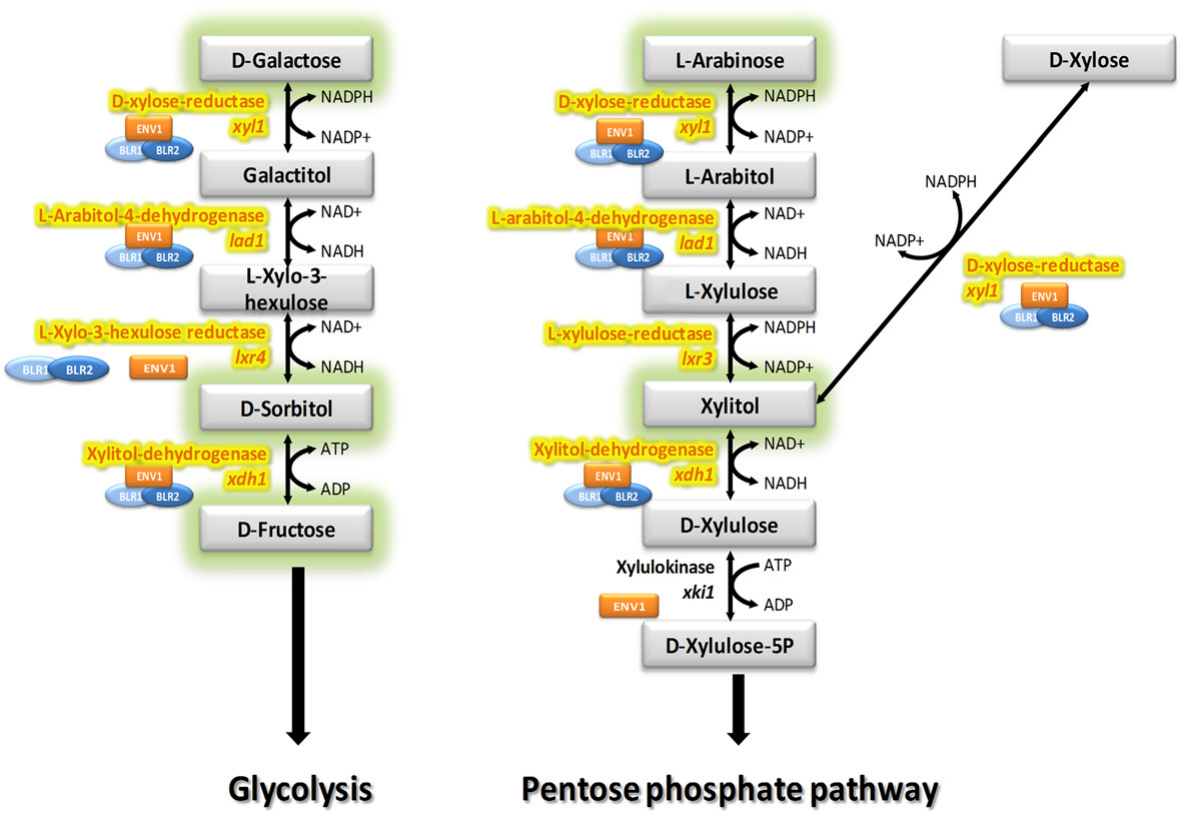
**Schematic representation of pentose and D-galactose catabolism in light.** Enzymes encoded by genes, which are positively regulated in light are highlighted in yellow. An influence of BLR1, BLR2 or ENV1 is indicated by their pictograms next to the enzyme. Metabolic intermediates of the pathway, on which growth is enhanced in light (BIOLOG assay) are represented with a green shadow. From the remaining intermediates, only D-xylose was also part of the assay, but no differences in growth were observed in wild-type or mutant strains. Galactitol, L-Xylo-3-hexulose, L-arabitol, D-Xylulose were not tested.

Transcription of all genes involved in catabolism of D-galactose and L-arabinose (Figure 7; [30]) except *xki1* showed a statistically significant increase in light in the wildtype (p-values <0.01). This indicates that the cascade starting from D-galactose or L-arabinose and D-xylose, respectively is positively affected by light during growth on cellulose.

Besides the Leloir pathway, an oxidoreductive pathway was identified in *T. reesei* and many of the enzymes involved are also part of the L-arabinose and D-xylose pathway [27,30]. The degradation of the hemicellulosic sugars D-xylose, L-arabinose and D-galactose starts with the same reaction: an NADPH-linked reduction by an aldose reductase XYL1 [28]. The next step, the oxidation of galactitol or L-arabitol is catalyzed by LAD1, the L-arabitol-4-dehydrogenase [49]. Recently it was shown that *lxr4*, encoding an L-xylo-3-hexulose reductase is the missing link in the oxidoreductive D-galactose catabolism in *T. reesei*[30]. The last step of the D-galactose degradation before glycolysis is the mediated by a NAD-xylitol dehydrogenase XDH1, which also catalyzes the conversion from xylitol to D-xylulose in the L-arabinose and D-xylose pathways [50]. The last phosphorylation step of the L-arabinose and D-xylose pathway before the substance enters the pentose phosphate pathway is presumably done by the gene product of *xki1*, a xylulose kinase and a homolog of *Aspergillus niger xkiA*[51].

Differential expression between constant light and constant darkness upon growth on cellulose was abolished upon lack of BLR1 and BLR2 for all genes investigated, except *xki1*, which does not show regulation by light in the wild-type (Figure 6). Transcript abundance predominantly drops to darkness levels in Δ*blr1* and Δ*blr2*. Hence, BLR1 and BLR2 are concluded to be necessary for positive regulation of this pathway in light.

For ENV1 the situation is more complex. In the absence of ENV1, differential expression between light and darkness is abolished for *lxr3* (which is enhanced to light-levels), *lad1* and *xdh1*. In contrast, the difference between transcript levels in light and darkness becomes even more pronounced for *lxr4* and *xyl1*, which is due to a strong upregulation of *lxr4* and of a considerable down-regulation of *xyl1* in light. Down-regulation of *xki1* in light upon lack of ENV1 results in differential expression of *xki1* in this strain, which was not observed in the wild-type. This effect is also one example, how lack of adaptation as mediated by ENV1, BLR1 or BLR2 could result in an increased number of genes differentially transcribed between light and darkness.

These results are in complete agreement with the results of the BIOLOG analysis, since lack of ENV1 causes decreased transcription of most of the enzymes in the pathway in light (Figures 5 and 7). Consequently, while the data for individual genes and growth on individual carbon sources for wild-type and mutant strains only show minor differences in dependence of light (mostly around 2fold, sometimes less), consistent regulation of growth and transcript abundance of the genes encoding enzymes of the oxidoreductive pentose and D-galactose pathway strongly indicates that the response of *T. reesei* to hemicellulose building blocks is modulated by photoreceptors in response to light.

A MEME search using the online version 4.9.0 (http://meme.nbcr.net/meme/cgi-bin/meme.cgi) did not reveal a common sequence in these promotors related to known light responsive motifs. According to the flat hierarchical network as proposed for photoreceptors *N. crassa*[52], the effect seen here is likely to be exerted indirectly. For *xyl1,* regulation by the cellulase and hemicellulase regulator XYR1 was shown [45,53]. We therefore screened all gene promoters of the pathway for XYR1-binding sites and only *xdh1* has a XYR1 binding site. As *xyr1*, similarly to *xdh1* and *xyl1* is positively regulated by ENV1 in light, this regulation might be mediated by XYR1.

### Lxr1 is part of a ENV1 regulated cluster in light

While initially assumed to be an L-xylulose reductase as it catalyzed the NADPH/NADP + specific reactions for L-xylulose/xylitol and for D-fructose/D-mannitol [54], LXR1 later turned out to be a mannitol dehydrogenase [55]. On D-mannitol, our growth analysis showed a slightly slower growth of the wild-type in light. Due to its additional activity on fructose [54], LXR1 could also be involved in the increased growth of the wild-type and the decreased growth in the *env1* mutant on this carbon source in light (Figure 5).

**Figure 8:**
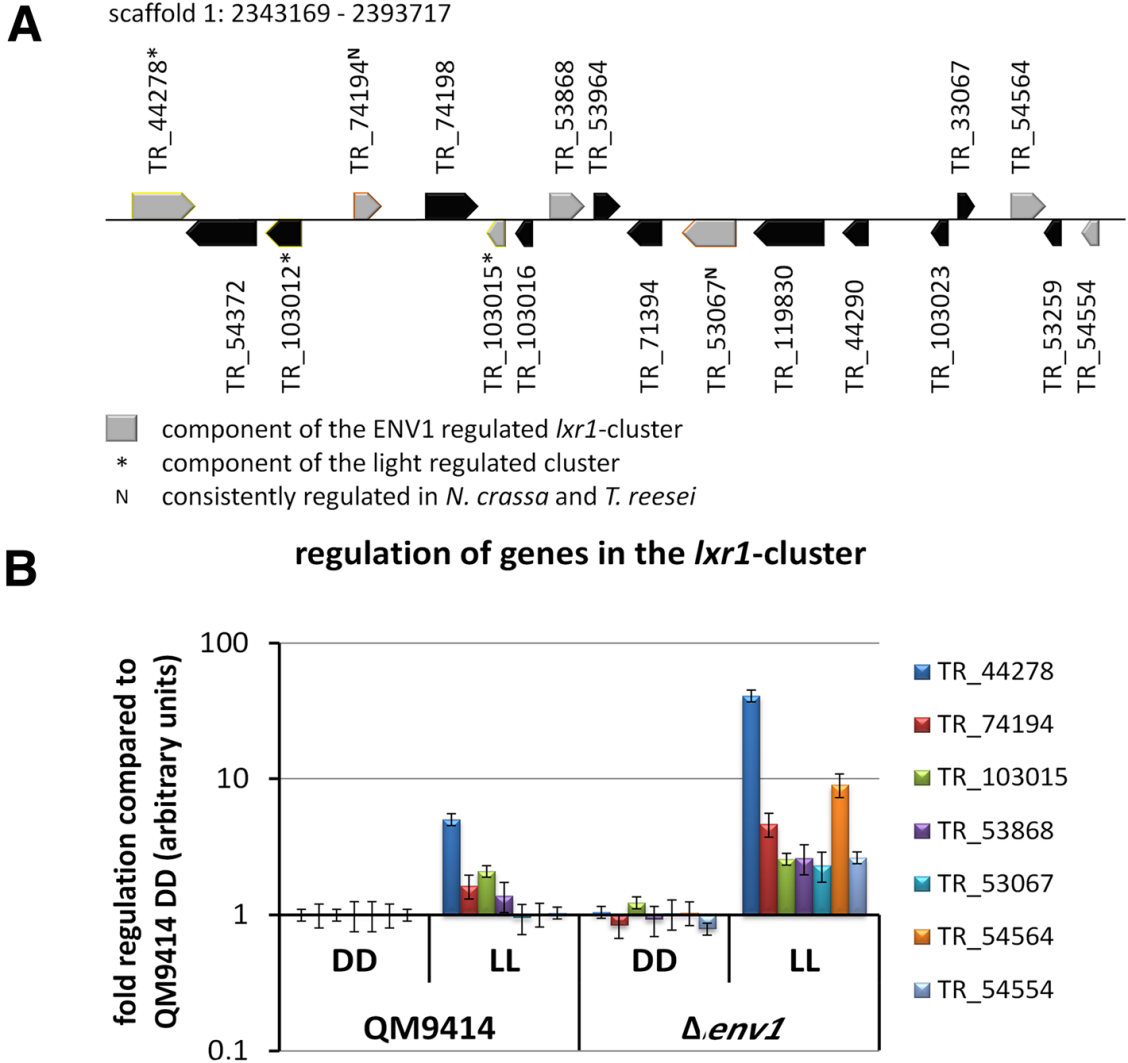
**Schematic representation of the ENV1-regulated *lxr1 *genomic cluster. (A)** The genomic region comprising the *lxr1* cluster is drawn to scale. Besides genes regulated by ENV1, further genes are located within this genomic region, which are given in black. Genes regulated by light on cellulose are given with yellow frames, those consistently regulated in *N. crassa* are shown with an orange frame. **(B)** Regulation of the *lxr1* cluster genes in QM9414 and ∆*env1* in light and darkness. No significant regulation of these genes was observed in the blr mutants.

Investigation of the genomic region around *lxr1* revealed that 9 genes in its genomic vicinity are regulated negatively (up to 40 fold) by ENV1 in light upon growth on cellulose and hence form a cluster (Figure 8A). BLR1 and BLR2 do not influence the regulation of the cluster. In contrast to the effect upon growth on mannitol, ENV1 has a strongly negative effect on *lxr1* and the genes in its cluster upon growth on cellulose (Figure 8B). The cluster is located at scaffold 1 between position 2343169 – 2393717 and comprises besides *lxr1* also a Zn-cluster transcription factor (TR_53067), a putative oligopeptide transporter (TR_44278), two probable old yellow enzyme (OYE) family flavin oxidoreductases (NADPH or NADH dependent; TR_103015 and TR_53868), a putative GTP cyclohydrolase (TR_54554) and a proline oxidase/dehydrogenase involved in conversion of proline for use as a carbon and nitrogen source (TR_54564). Two of these genes, *lxr1* and TR_103015 have homologues in *N. crassa* (NCU09041 and NCU04452), which are responsive to light ( [18]). However, neither these nor the other members of this cluster are syntenic in *N. crassa*.

Within the borders of the ENV1-regulated *lxr1*-cluster, a light regulated cluster starts (Figure 8A), which comprises TR_103015, TR44278, as well as a taurine dioxigenase involved in sulphur metabolism (TR_103012) and additionally contains a GATase1-like (glutamine-amidotransferase type 1) domain containing peptidase (TR_103039), which is located outside the *lxr1* cluster.

### Consistent targets of photoreceptors in *N. crassa* and *T. reesei*

Previous studies indicated at least in part comparable regulation of cellulase gene expression by light and photoreceptors in *T. reesei* and *N. crassa*[13,14,22,25]. We were hence interested, whether the regulatory targets of photoreceptors in these two fungi are similar. Therefore, we re-analyzed the transcriptome data for *N. crassa* wild-type as well as Δ*wc-1*, Δ*wc-1* and Δ*vvd* upon growth on cellulose in light ( [25]; GEO Accession number: GSE32871) for significant 2fold differential regulation in order to be comparable with our data. 609 genes were found to be differentially regulated in one or more of the *N. crassa* photoreceptor mutants and are hence photoreceptor targets in light. 369 of these genes have reciprocal best hits (p-value for blast analysis set to 1e-05) in *T. reesei* and are therefore likely homologues. 55 genes were identified as consistent targets of one or more photoreceptors in *N. crassa* and *T. reesei* (Additional file 10). In agreement with previous findings, these genes were enriched in functions of metabolism (p-value 1.63e-05), particularly C-compound and carbohydrate metabolism (p-value 1.71 e-04) and sugar, glucoside, polyol and carboxylate metabolism (p-value 1.56e-03). Additionally, functions in C-compound and carbohydrate transport (p-value 2.38e-05) and glycolysis and gluconeogenesis (p-value 4.86e-03) were enriched. Among the consistent photoreceptor targets are further five glycoside hydrolases (TR_120229 (*xyn3*), TR123818 (*xyn2*), TR_123989 (*cbh1*), TR_72526 (*glr1*), TR_72567 (*cbh2*)), one carbohydrate esterase (TR_72072) and two genes involved in sexual development (TR_104292, TR_123697) including the gene encoding the alpha-type peptide pheromone precursor *ppg-1* which was shown to impact cellulase regulation in *N. crassa*[25]. Two putative sugar transporters (TR_76800 and TR_106556) and one putative carboxylic acid transporter (TR_121441) were also among these genes. Although functions in sulphur and amino acid metabolism were not enriched among these 55 genes, two putative methionine synthases (TR_121820 and TR_3823) as well as one methionine permease (TR_77969) were consistently regulated. Moreover, two transcription factors were found to be consistent targets: TR_120715, which has not yet been characterized and TR_53067, the homologue of *tah-2*, which is involved in conidiophore development [56] and shows increased transcript levels upon growth on miscanthus [57] in *N. crassa*. TR_53067 is part of the *lxr1* cluster described above and intriguingly, also *lxr1* itself is among the consistently regulated genes. However, the well characterized cellulase regulators of *T. reesei* and *N. crassa*, such as *xyr1*, *cre1*, *clr-1* or *clr-2* are not among the consistently regulated genes.

## Discussion

In this study we investigate the light signalling machinery of *T. reesei* at a genome wide level upon growth on cellulose, which is the closest defined carbon source to what *T. reesei* encounters in its natural habitat. We extend previous knowledge on the function of photoreceptors, which was mainly done on glucose and in constant light, with investigating the effect of the photoreceptors in darkness. This enabled us to study both differential gene regulation between growth in light and darkness (termed light responsiveness here) and investigation of regulatory targets (differential regulation compared to wild-type) of BLR1, BLR2 and ENV1 in light and darkness. Although only few genes are regulated by the photoreceptors in darkness, a clear role in carbon metabolism and transport functions could be detected in darkness. These functions resemble also the targets in light, indicating that regulation by photoreceptors is not strictly light dependent.

One of the most intriguing and puzzling findings of this study was the increased number of differentially transcribed genes in the photoreceptor mutants, as we rather expected the opposite effect. A similar phenomenon was observed for mutants in the G-protein pathway [10]. One explanation for the reason that so many genes are light-regulated in the photoreceptor mutants might be a lack of light adaptation in the photoreceptor mutants: in the comparison between growth in constant darkness and in constant light, many transcripts might appear unchanged due to adaptation to light. However upon deletion of BLR1/BLR2 or ENV, both light induction and adaptation are assumed to be lost, which would cause decreased/altered transcript levels in the mutants. Indeed, evaluation of the regulatory targets of BLR1, BLR2 and ENV1 revealed the highest numbers of regulated transcripts to be downregulated in the mutant strains in constant light (Additional file 4, Additional file 5 and Additional file 6), which supports this hypothesis.

While we cannot provide a mechanistic explanation yet, our working hypothesis currently involves a higher order regulation to be targeted by the photoreceptors such as chromatin remodelling, which was shown to be impacted by light and the clock [58,59]. This would explain the wide-spread effect we see and it would be in agreement with a rather tight regulation in the wild-type. Nevertheless, also such a mechanism would require an additional, so far unidentified factor to transmit the light signal in the absence of photoreceptors.

Recently, investigation of the effect of photoreceptors on cellulase gene expression in *N. crassa* revealed that this process is subject to photoadaptation in this fungus. Additionally, the known cellulase repressor gene *ace1*, the carbon catabolite repressor gene *cre1* and the cross pathway control protein encoding *cpc1*, which is involved in response to amino acid starvation response, were found to be regulated by photoreceptors in *N. crassa*[25]. In *T. reesei* however, the situation appears to be different. We could identify the cellulase and hemicellulase regulator gene *xyr1* to be a target of BLR1 and ENV1 on cellulose, whereas *N. crassa xyr-1* is not regulated by photoreceptors. Also the *T. reesei* homologues of *clr-1* and *clr-2*[60], two recently identified *N. crassa* cellulase regulators, (TR_26163 and TR_27600) are subject to regulation by photoreceptors, although they are not light regulated in the wild-type. Additionally, neither *ace1* nor *cre1* or *cpc1* are among the targets of the light signalling machinery in *T. reesei*. Hence, despite largely similar global processes to be targeted by BLR1, BLR2 and ENV1, such as carbon-, amino acid- and sulphur metabolism, the regulation of the involved pathways seems to be achieved differently in *N. crassa* and *T. reesei*. This finding is also in agreement with consistent regulation of metabolic genes but not the respective known regulators between *N. crassa* and *T. reesei* in light. Also the effects on glycogen metabolism observed in *N. crassa* were not obvious for *T. reesei*. However, the general influence of photoreceptors on carbon and amino acid metabolism as well as its assumed correlation with cellulase gene expression [25,34,61] is clearly conserved in *T. reesei* and *N. crassa*.

The results of this study illustrate an important phenotypic characteristic of strains lacking ENV1. These strains show severely reduced growth rate and conidiation, both in liquid media and on plates in light [13,22,24]. This defect is clearly reflected in the strongly altered transcriptome of this strain in light compared to darkness. In the absence of ENV1, considerable alterations involving both positive and negative regulation of metabolic genes – especially those in carbon and amino acid metabolism – as well as genes for substrate transport and energy metabolism occurs. Hence ENV1 is of crucial importance for adjustment of metabolism to changing light conditions. So far we could not identify the reason for the diminished growth rate of Δ*env1* under the conditions we used and hence effects besides metabolic imbalance may influence the phenotype of this strain. Additionally, it was found that growth rate does not necessarily correlate with production of hydrolytic enzymes in *T. reesei* ( [10] and references therein). Despite its slow growth, specific cellulase activity secreted into the medium by Δ*env1* is several fold increased compared to wild-type (M. Schmoll, unpublished). We conclude that ENV1 is an essential regulator/signal transmitter for adjustment of growth rate to environmental conditions in light. This function, however, is most important on cellulose, as the BIOLOG analysis on numerous carbon sources (Figure 5) showed that the light dependent growth defect of Δ*env1* does not occur on every carbon source. Therefore, it can be assumed that the function of ENV1 in light is most critical upon growth on cellulose, but less important for growth in the presence of other carbon sources in light.

To complement our transcriptome analysis for growth on cellulose, we compared the growth patterns on different carbon sources in light and darkness for wild-type and an *env1* non functional strain [13,22], because of the strong effect of ENV1 on light dependent gene regulation. Earlier analysis had indicated that the degradation of different carbon sources is regulated in a light dependent manner and that ENV1 is involved in the transfer of the positive light signal in many cases [23]. Our re-evaluation and cluster analysis showed that especially intermediates of the oxidoreductive D-galactose and L-arabinose pathway (Figure 7), which is also important for utilization of lactose, are among the carbon sources on which growth in the wild-type and the *env1* mutant were most clearly influenced. Due to the ability of *T. reesei* to grow and express cellulases on lactose, the degradation of lactose is very well investigated [28]. In eukaryotes lactose is first cleaved to glucose and galactose and galactose is further converted into glucose 6-phosphate by enzymes of the Leloir pathway. Together with arabinose, also catabolism of galactose is involved in utilization of hemicellulose building blocks [47]. As for the most part, plant cell wall degrading enzymes are coregulated in *T. reesei*[43], this is in accordance with our finding that the genes encoding the enzymes operative in these pathways [30] are expressed upon growth on cellulose. These findings are in agreement with the hypothesis that *T. reesei* senses the presence of plant cell wall material in its environment by detection of building blocks of cellulose and hemicellulose. Interpretation of the respective signals is subsequently adjusted to the requirements in light and darkness by BLR1, BLR2 and ENV1.

## Conclusions

In summary, we showed that BLR1, BLR2 and ENV1 exert important metabolic functions, not only in light, but also in darkness. Lack of components of the light signalling machinery causes considerably increased light responsiveness of transcript levels, likely caused by a light-dependent, positive effect on output pathways. Thereby, the photoreceptors BLR1 and BLR2 do not exclusively act as a complex, but additionally have individual targets. This finding also applies to ENV1, for which the transcriptome pattern indicates a sizable amount of independent targets in light. Interestingly, the targets of the light response machinery also include the catabolic enzymes necessary for degradation of hemicellulose building blocks i. e. the D-galactose and pentose pathway and *lxr1*, which adds a new aspect to light dependent gene regulation on cellulose. Comparison with transcript profiles of *N. crassa* indicates a conserved adjustment of metabolic pathways in light by photoreceptors, but also different regulatory mechanisms applied in order to achieve this effect.

## Methods

### Strains and culture conditions

*Trichoderma reesei* strain QM9414 (ATCC 26921) was used as the parental strain, and the recombinant strains ∆*env1*, ∆*blr1* and ∆*blr2*[13], were analysed throughout this study.

For the inoculum, strains were grown on malt extract medium for 14 days in constant darkness until sporulation in order to avoid interference of random light pulses. For cultivation, strains were grown in 1 L shake flasks at 28°C on a rotary shaker (200 rpm) in Mandels-Andreotti minimal medium [62], supplemented with 0.1% (w/v) peptone to induce germination using 1% (w/v) microcrystalline cellulose (#1402; SERVA, Heidelberg, Germany) as carbon source. Strains were grown for 72 hours in constant darkness (indicated with DD) or constant light (LL, 25 μmol photons m^-2^ s^-1^; 1800 lux). Harvesting of dark grown cultures was done under safe-red-light (darkroom lamp, Philips PF712E, red, E27, 15 W). Strains were grown in two biological replicates and at least two biological replicates were used in the analyses described below.

### Nucleic acid isolation and manipulation

For isolation of nucleic acids, the mycelium was filtered, briefly washed with medium containing no carbon source and frozen in liquid nitrogen. Total RNA was isolated as described elsewhere [24]. The concentration was measured with a Nanodrop ND-1000 spectrophotometer (PEQLAB, Erlangen, Germany). Total RNA was treated with DNase I (Thermo Fisher / Fermentas, Vilnius, Lithuania) and purified using the RNeasy Plant Mini Kit (QIAGEN, Hilden, Germany). The quality of total RNA was evaluated using the Experion Automated Electrophoresis System (Bio-Rad, Hercules, USA) and the Experion RNA StdSens Analysis Kit (Bio-Rad). The treshold for minimum quality for use in our experiments was set to RQI > 7.

### Quanitative reverse transcription PCR and microarray analysis

cDNA for microarray experiments was obtained by reverse-transcribing five μg of purified total RNA with RevertAid-H^-^ First Strand cDNA Synthesis Kit (Thermo Fisher / Fermentas) using Random Hexamer Primers following the manufacturer’s instructions. cDNA for qRT-PCR was obtained similarly, except for the use of oligo-d(T)-primers instead of the Random Hexamer Primers. iQ SYBR Green supermix (Bio-rad) and the IQ5 ICycler system (Bio-rad) were used for qRT-PCR, The open source software REST (relative expression software tool) was applied for data analysis and evaluation of significant differential expression between different strains in light and darkness [63]. The experiments were done in technical triplicates from at least two independent biological replicates (for primer sequences and PCA analysis of replicate datasets see Additional file 2: Table S3 and Figure S2). For normalization of the qRT-PCR data the ribosomal gene *rpl6e* was used, as it shows robust constitutive transcript levels on cellulose in light and darkness [10,24].

The gene expression full service for custom arrays as provided by Roche-NimbleGen (Roche-NimbleGen, Madison, USA) was used for microarray analysis with two biological replicates. Oligonucleotide arrays were used as described in [10]. Data analyzed in this study are deposited at NCBI Gene Expression Omnibus with accession numbers GSE36448, GSM683732, 683733, 683734 and 683735.

Microarray data analysis was done by using PARTEK Genomics Suite 6.6 (PARTEK Inc., St. Louis, Missouri, USA), which uses ANOVA for evaluation of statistically significant differentially expressed genes. As threshold for the significant regulation of a gene a twofold transcriptional difference between light and darkness (i.e. light responsiveness) or between a mutant strain and the wildtype (i.e. targets) was applied. For significance the combined p-value for significant regulation due to different light conditions and different strains was set to <0.1. No adjustment has been made for multiple hypothesis testing and p-values reported are suggestive of an association. Hierarchical clustering was done using the open source software HCE 3.5 with default settings [64]; http://www.cs.umd.edu/hcil/hce). Genomic cluster analysis was performed using the open source software REEF [65,66]

Results were analyzed using the community annotation including GO (Gene Ontology) classifications from the *T. reesei* genome database v2.0 provided by JGI (http://genome.jgi-psf.org/Trire2/Trire2.home.html) and revised annotations from [10].

## Competing interests

The authors declare that they have no competing interests.

## Authors’ contributions

DT performed the experiments, interpreted the results and drafted the manuscript. MS conceived of the study, participated in bioinformatics analysis, interpretation of results and wrote the final version of the manuscript. Both authors read and approved the final manuscript.

## Supplementary Material

Additional file 1 **Light responsiveness of transcript abundance as influenced by BLR1, BLR2 or ENV1.** Genes at least two-fold up- or downregulated in light compared to darkness in QM9414 and deletion strains ∆*blr1*, ∆*blr2* and ∆*env1*. Click here for file

Additional file 2 **Regulation by ENV1 in darkness.** Genes specifically upregulated in ∆*env1* in darkness as revealed by hierarchical cluster analysis of transcript profiles in QM9414 and deletion strains ∆*blr1*, ∆*blr2* and ∆*env1*. Click here for file

Additional file 3 **Supporting information. Figure S1.** Co-regulation of *cel6a*/*cbh2* (data obtained from microarrays) and *cel7a*/*cbh1* (data obtained from qPCR) in QM9414 and ∆*blr1*, ∆*blr2*, ∆*env1*, ∆*gnb1*, ∆*gng1* and ∆*phlp1*. **Table S1.** Regulation of glycoside hydrolase genes in QM9414 and the deletion strains ∆*env1*, ∆*blr1* and ∆*blr2* and comparison with regulations in ∆*phlp1*, ∆*gnb1* and ∆*gng1*. **Table S2.** Genes coregulated with *cbh2/cel6a* in QM9414 and the deletion strains ∆*env1*, ∆*blr1* and ∆*blr2.***Table S3.** Sequences of oligonucleotides used in this study. Click here for file

Additional file 4 **Regulatory targets of ENV1.** Genes at least two-fold up- or downregulated in ∆*env1* compared to QM9414 in light (LL) and darkness (DD). Click here for file

Additional file 5 **Regulatory targets of BLR1.** Genes at least two-fold up- or downregulated in ∆*blr1* compared to QM9414 in light (LL) and darkness (DD). Click here for file

Additional file 6 **Regulatory targets of BLR2.** Genes at least two-fold up- or downregulated in ∆*blr2* compared to QM9414 in light (LL) and darkness (DD). Click here for file

Additional file 7 **Overlapping targets of ENV1, BLR1 and BLR2.** Genes at least two-fold up- or downregulated in ∆*env1*, ∆*blr1* and ∆*blr2* compared to QM9414 in light (LL) and darkness (DD). For an overview see Figure 4, which shows the number of genes comprised in each sheet of the file. Click here for file

Additional file 8 **Targets of BLR1 and BLR2 (BLR complex) versus ENV1.** Genes at least two-fold downregulated in ∆*blr1* and ∆*blr2* compared to QM9414 in light but upregulated in ∆*env1* and genes at least two-fold up- or downregulated in ∆*blr1* and ∆*blr2* compared to QM9414 but not in ∆*env1* in light and darkness. For an overview see Figure 4, which shows the number of genes comprised in each sheet of the file. Click here for file

Additional file 9 **Individual targets of ENV1.** Genes at least two-fold differentially regulated in ∆*env1* compared to QM9414 in light and darkness, which are not targets of BLR1 or BLR2. For an overview see Figure 4, which shows the number of genes comprised in each sheet of the file. Click here for file

Additional file 10 **Genes consistently regulated in *N. crassa *** **and *T. reesei *** **in one or more photoreceptor mutant strains upon growth on cellulose in light.** Click here for file
