# Supplementary material for: Targets of light signalling in Trichoderma reesei
Source: BMC Genomics. 2013 Sep 26;14:657. doi: 10.1186/1471-2164-14-657 (PMC3831817; doi:10.1186/1471-2164-14-657)
Supplement: Additional file 2 — Regulation by ENV1 in darkness. Genes specifically upregulated in ∆env1 in darkness as revealed by hierarchical cluster analysis of transcript profiles in QM9414 and deletion strains ∆blr1, ∆blr2 and ∆env1. [file 1471-2164-14-657-S2.pdf]

Additional file 2

Figure S1 - Co-regulation of *cel6a*/*cbh2* (data obtained from microarrays) and *cel7a*/*cbh1* (data obtained from qPCR) in QM9414 and Δ*blr1*, Δ*blr2*, Δ*env1*, Δ*gnb1*, Δ*gng1* and Δ*phlp1*.

Figure S2 – PCA analysis of replicate datasets of different mutants and conditions.

Table S1 - Regulation of glycoside hydrolase genes in QM9414 and the deletion strains Δ*env1*, Δ*blr1* and Δ*blr2* and comparison with regulations in Δ*phlp1*, Δ*gnb1* and Δ*gng1*.

Table S2 – Genes coregulated with cbh2/cel6a in QM9414 and Δ*blr1*, Δ*blr2* and Δ*env1*.

Table S3 - Sequences of oligonucleotides used in this study.

Supplementary Figures

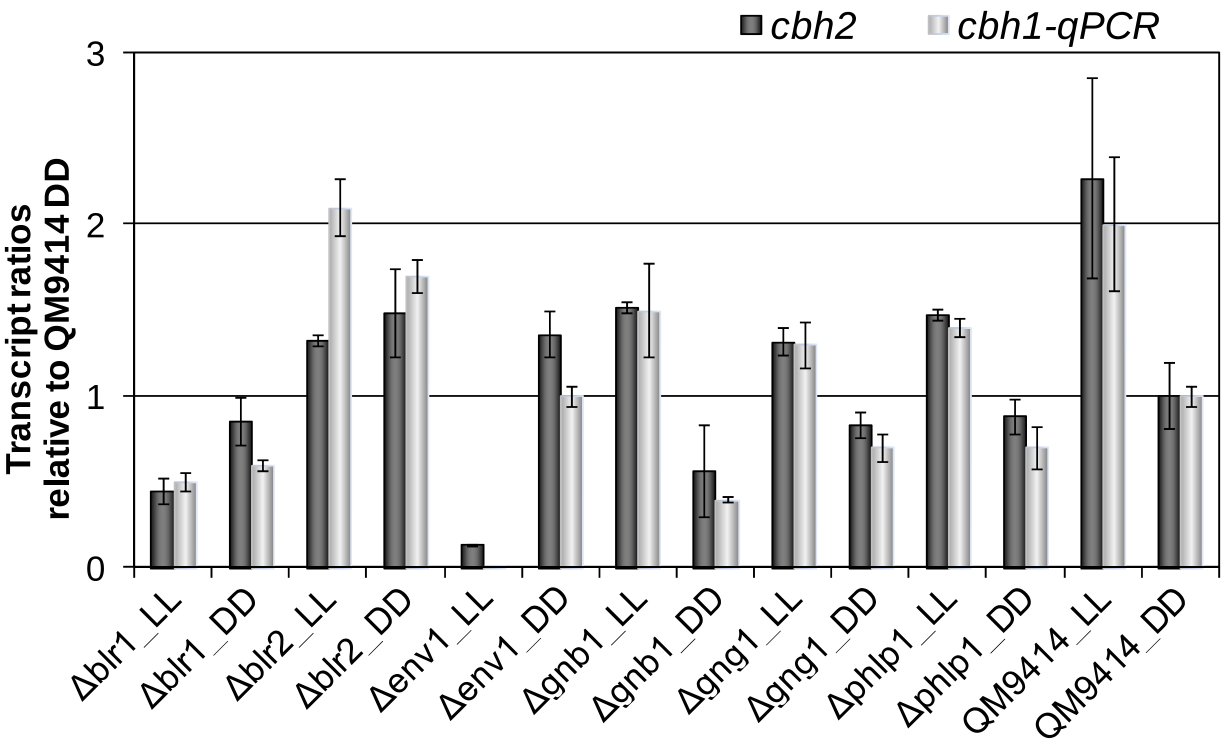

Figure S1 - Co-regulation of *cel6a*/*cbh2* (data obtained from microarrays) and *cel7a*/*cbh1* (data obtained from qRT-PCR) in QM9414 and Δ*blr1*, Δ*blr2*, Δ*env1*, Δ*gnb1*, Δ*gng1* and Δ*phlp1*. Strains were grown on 1 % (w/v) microcrystalline cellulose as carbon source for 72 hours in constant light (LL) or darkness (DD). Δ*gnb1*, Δ*gng1* and Δ*phlp1* were included in the analysis as additional controls for coregulation of *cbh1* and *cbh2* in different strains.

Supplementary Figure S2 – PCA analysis of replicate datasets of different mutants and conditions.

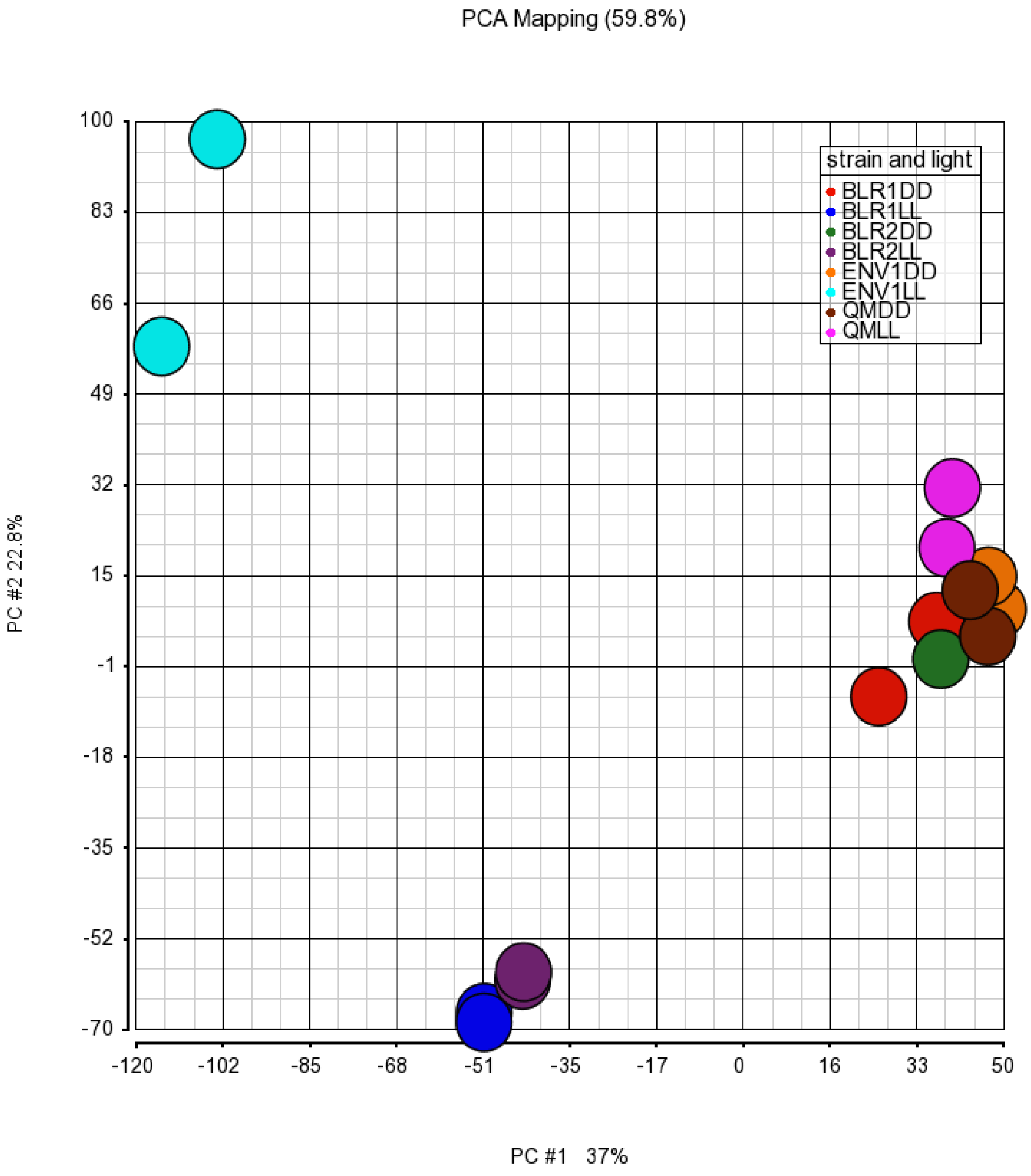

Supplementary Tables

Supplementary Table S1 Regulation of glycoside hydrolase genes in QM9414 and the deletion strains Δ*env1* , Δ*blr1* and Δ*blr2* and comparison with regulations in Δ*phlp1* , Δ*gnb1* and Δ*gng1*.

If all genes of a glycoside hydrolase family are potentially light responsive, members of this family are given in bold

GENE_ID	Gene name	Description	Specification	Glycoside Hydrolase Family	all*	here**	upregulation							downregulation							PhLP1-GNB1-GNG1 target	ENV1-BLR1-BLR2 target	shared target
							WT	Δ*phlp1*	Δ*gnb1*	Δ*gng1*	Δ*env1*	Δ*blr1*	Δ*blr2*	WT	Δ*phlp1*	Δ*gnb1*	Δ*gng1*	Δ*env1*	Δ*blr1*	Δ*blr2*			
**22197**	**cel1B**	**b-glucosidase CEL1b (GenBank acc. No. AAP57758)**		**1**	**2**	**2**												**X**					
**120749**	**cel1A**	**b-glucosidase (GenBank acc. No. BAA74959)**	**cellulolytic enzyme**	**1**	**2**	**2**			**X**									**X**					
**120229**		**candidate xylanase**	**hemicellulolytic enzyme**	**10**	**1**	**1**	**X**	**X**	**X**	**X**								**X**					
**57179**		**candidate glycosidase distantly related to unsaturated rhamnogalactuonyl hydrolase**		**105**	**1**	**1**												**X**	**X**				
**74223**	**xyn1**	**xylanase 1 (EC 3.2.1.8)**	**hemicellulolytic enzyme**	**11**	**3**	**3**									**X**	**X**							
**112392**		**candidate xylanase**	**hemicellulolytic enzyme**	**11**	**3**	**3**										**X**		**X**			**X**		
**123818**	**xyn2**	**xylanase 2 (EC 3.2.1.8)**	**hemicellulolytic enzyme**	**11**	**3**	**3**	**X**	**X**	**X**	**X**								**X**				**X**	
**77284**		**candidate b-glycosidase distantly related to endoglucanase**		**12**	**2**	**2**												**X**					
**123232**	**cel12A**	**endoglucanase**	**cellulolytic enzyme**	**12**	**2**	**2**	**X**	**X**	**X**	**X**								**X**	**X**				
**57128**		**candidate glycogen-debranching enzyme**		**13**	**5**	**5**									**X**		**X**	**X**	**X**			**X**	
**59578**		**candidate oligo-1,6-glucosidase**		**13**	**5**	**5**										**X**							
**105956**		**candidate a-amylase**		**13**	**5**	**5**												**X**					
**108477**		**candidate oligo-1,6-glucosdiase**		**13**	**5**	**5**												**X**					
**123368**		**candidate glycogen branching enzyme**		**13**	**5**	**5**												**X**					
**65333**		**candidate a-glycosidase distantly related to glucoamylase and a,a-trehalase**		**15**	**1**	**1**			**X**		**X**												
38536		candidate b-glycosidase related to b-1,3-1,4-glucanase		16	16	13										X		X		X	**X**		
41768		candidate b-glycosidase related to cell-wall modifying enzymes		16	16	13									X	X		X		X	**X**	**X**	**X**
49274		candidate b-glycosidase related to b-1,3-1,4-glucanase		16	16	13								X	X	X	X						
50215		candidate b-glycosidase distantly related to endo-1,3-b-D-glucanase		16	16	13			X									X					
55886		candidate b-glycosidase related to b-1,3-1,4-glucanase		16	16	13									X	X		X	X	X	**X**		
65406		candidate b-glycosidase related to cell-wall modifying enzymes		16	16	13										X							
66843		candidate b-glycosidase related to cell-wall modifying enzymes		16	16	13												X					
70542		candidate b-glycosidase distantly related to b-1,3-1,4-glucanase/lichenase		16	16	13					X						X						
71399		candidate b-glycosidase distantly related to b-1,3-glucanase		16	16	13											X						
73101		candidate b-glycosidase distantly related to b-1,3-1,4-glucanase		16	16	13									X	X	X	X		X	**X**	**X**	**X**
121294		candidate b-glycosidase realted to b-1,3-1,4-glucanase		16	16	13					X											**X**	
122511		candidate endo-1,3(4)-b-glucanase		16	16	13			X														
123726		candidate endo-1,3(4)-b-glucanase		16	16	13										X			X	X			
39942		candidate b-glycosidase distantly related to exo-1,3-glucanase		17	4	3					X												
49193		candidate b-glycosidase distantly related to exo-1,3-glucanase		17	4	3												X					
66792		candidate b-glycosidase distantly related to exo-1,3-glucanase		17	4	3												X					
2735	chi18-6	chitinase; fungal subgroup A chitinase		18	20	16										X	X			X			
56448	chit18-11	chitinase; fungal subgroup A chitinase		18	20	16										X		X					
56894		candidate b-glycosidase related to chitinase; fungal subgroup C chitinase		18	20	16													X				
59082	chi18-2	candidate b-glycosidase related to chitinase; fungal subgroup A chitinase		18	20	16									X		X	X					

* total number of genes belonging to this GH family in *T. reesei*

** number of genes of this GH family being potentially regulated by light, as described in this study

GENE_ID	Gene name	Description	Specification	Glycoside Hydrolase Family	all*	here**	upregulation							downregulation							PhLP1-GNB1-GNG1 target	ENV1-BLR1-BLR2 target	shared target
							WT	Δ*phlp1*	Δ*gnb1*	Δ*gng1*	Δ*env1*	Δ*blr1*	Δ*blr2*	WT	Δ*phlp1*	Δ*gnb1*	Δ*gng1*	Δ*env1*	Δ*blr1*	Δ*blr2*			
59791	chi18-15	candidate chitinase related to bacterial chitinases, does not belong to a fungal subgroup		18	20	16									X	X							
62704	chi18-3	chitinase; fungal subgroup A chitinase		18	20	16										X							
65162		candidate b-glycosidase related to chitinase		18	20	16										X		X					
71245	chi18-1	chitinase, precursor protein, extracellular, contains chitin binding domain, type 1		18	20	16									X	X	X	X	X	X	**X**	**X**	**X**
72339	chi18-9	candidate b-glycosidase related to chitinase; CHI18-9 (GenBank acc. No. CDAA05857); fungal subgroup C chitinase		18	20	16			X		X												
80833	chi18-5	chitinase, fungal subgroup A chitinase; known as ECH42 in other *Trichoderma* species		18	20	16		X			X												
81598	chi18-7	chitinase; fungal subgroup A chitinase		18	20	16												X					
108346	chi18-8	candidate b-glycosidase related to chitinase; CHI18-8 (GenBank acc. No. DAA05856); fungal subgroup C chitinase		18	20	16									X	X	X		X	X	**X**		
110317	chi18-17	chitinase; C-terminal CBM1 module; fungal subgroup B chitinase		18	20	16										X		X		X			
119859	chi18-13	chitinase; fungal subgroup B chitinase		18	20	16										X		X					
121355		candidate b-glycosidase distantly related to chitinase		18	20	16					X												
124043	chi18-14	chitinase; fungal subgroup B chitinase		18	20	16									X	X	X	X	X	X			
**5836**		**candidate b-mannosidase**		**2**	**7**	**7**																	
**57857**		**candidate b-mannosidase**		**2**	**7**	**7**												**X**					
**59689**		**candidate b-mannosidase**		**2**	**7**	**7**									**X**		**X**						
**62166**		**candidate b-mannosidase**		**2**	**7**	**7**			**X**									**X**					
**69245**		**candidate b-mannosidase**		**2**	**7**	**7**	**X**																
**76852**		**candidate b-glycosidase distantly related to b-galactosidase**		**2**	**7**	**7**					**X**												
**77299**		**exo-b-D-glucosaminidase GLS93 (GenBank acc. No. BAD99604)**		**2**	**7**	**7**												**X**					
105931		candidate b-glycosidase distantly related to N-acetyl-b-D-glucosaminidase		20	3	1									X	X		X	X	X			
**109278**		**candidate b-glycosidase distantly related to lysozyme**		**24**	**1**	**1**					**X**					**X**							
**103458**		**candidate b-glycosidase related to lysozyme**		**25**	**1**	**1**										**X**		**X**		**X**			
27219		candidate a-glycosidase related to a-galactosidase		27	8	6									X	X	X		X	X	**X**	**X**	**X**
27259		candidate a-glycosidase related to a-galactosidase		27	8	6										X							
55999		candidate a-galactosidase		27	8	6												X	X				
59391		candidate a-galactosidase		27	8	6									X		X						
65986		candidate a-galactosidase		27	8	6									X	X	X	X		X	**X**		
75015		candidate a-glycosidase related to a-galactosidase		27	8	6										X							
70186		candidate a-glycosidase related to exo-polygalacturonase		28	4	3											X				**X**		
103049		candidate a-glycosidase related to exo-polygalacturonase		28	4	3									X						**X**		
122780		candidate a-glycosidase related to exo-polygalacturonase		28	4	3												X					
46816	cel3D	b-glucosidase (GenBank acc. No. AAP57759)	cellulolytic enzyme	3	13	9			X									X					
47268		candidate b-glucosidase		3	13	9					X	X											
58450		cadidate b-glycosidase distantly related to a-L-arabinofuanosidase / b-xylosidase		3	13	9												X					
69557		candidate b-glycosidase related to b-N-acetylglucosaminidase		3	13	9												X					
76227	cel3E	b-glucosidase (genBank acc. No. AAP57760)	cellulolytic enzyme	3	13	9											X	X					
76672	cel3A	b-glucosidase		3	13	9												X	X				
82227	cel3C	b-glucosidase (GenBank acc. No. AAP57756)		3	13	9												X					
108671		candidate b-glycosidase related to b-xylosidase		3	13	9													X			**X**	
121127	bxl1	b-xylosidase (GenBank acc. No.CAA93248)		3	13	9	X		X									X					
**3094**		**candidate b-1,6-glucanase; similar to *T. harzianum* BGN16.3 (GenBank acc no Q8J0I9)**		**30**	**2**	**2**									**X**	**X**	**X**	**X**	**X**	**X**	**X**	**X**	**X**

* total number of genes belonging to this GH family in *T. reesei*

** number of genes of this GH family being potentially regulated by light, as described in this study

GENE_ID	Gene name	Description	Specification	Glycoside Hydrolase Family	all*	here**	upregulation							downregulation							PhLP1-GNB1-GNG1 target	ENV1-BLR1-BLR2 target	shared target
							WT	Δ*phlp1*	Δ*gnb1*	Δ*gng1*	Δ*env1*	Δ*blr1*	Δ*blr2*	WT	Δ*phlp1*	Δ*gnb1*	Δ*gng1*	Δ*env1*	Δ*blr1*	Δ*blr2*			
69736		candidate b-1,6-glucanase		30	2	2										X		X					
60085		candidate a-glycosidase distantly related to a-glucosidase		31	4	4												X					
69944		candidate a-xylosidase		31	4	4	X		X									X					
82235		candidate a-glucosidase (required for normal cell wall synthesis)		31	4	4		X	X	X								X					
121351		a-glucosidase II from RutC30(GenBank acc. No. AAU87850.1)(EC 3.2.1.-)		31	4	4												X					
124016	agl2	a-galactosidase(GenBank acc. No. CAA93245) (EC 3.2.1.22)		36	2	1			X		X												
120676		candidate neutral trehalase		37	2	2												X					
123226		candidate trehalase		37	2	2					X												
3196		candidate a-mannosidase		38	1	1												X					
73102		candidate b-glycosidase		39	1	1									X			X	X		X		
3739		candidate b-xylosidase	hemicellulolytic enzyme	43	2	2			X										X				
68064		candidate b-glycosidase distantly related to b-xylosidase / a-L-arabinofuranosidase	hemicellulolytic enzyme	43	2	2												X					
49976	cel45A	endoglucanase V EGL5 (CEL45A)	cellulolytic enzyme	45	1	1												X	X				
79960		candidate a-mannosidase		47	8	1												X					
56996	man5A	mannan endo-1,4-b-mannosidase (EC 3.2.1.78); C-terminal CBM1 module		5	11	7	X		X									X	X				
64375		candidate exo-b-1,3-glucanase		5	11	7										X		X					
64906		candidate b-1,6-glucanase; similar to *T. harzianum* B16-2 (GenBank acc. No. CAA55789) and *T. virens* BGN3 (AAL84696)		5	11	7									X	X	X	X	X	X	X	X	X
69276		candidate b-glycosidase distantly related to xylanase		5	11	7	X	X	X	X									X				
71554		candidate b-glycosidase distantly related to b-mannanase		5	11	7										X							
111849	xyn4	xylanase 4		5	11	7			X									X					
120312	cel5A	endo-1,4-glucanase EGL2	cellulolytic enzyme	5	11	7	X		X									X	X				
123283	abf1	a-L-arabinofuranosidase ABF1 (GenBand acc. No. CAA93243)	hemicellulolytic enzyme	54	2	2					X												
55319		candidate a-L-arabinofuranosidase	hemicellulolytic enzyme	54	2	2	X	X		X								X				X	
121746		candidate b-1,3-exoglucanase; related to GLUC78 (GenBank acc. No. AAF80600) of T. atroviride		55	6	6												X					
54242		candidate b-glycosidase related to b-1,3-glucanase		55	6	6										X		X					
56418		candidate b-1,3-exoglucanase		55	6	6									X	X	X	X	X	X	X	X	X
70845		candidate b-1,3 glucanase; similar to *T. virens* BGN1 (GenBank acc. No. AAL84694)		55	6	6										X		X					
73248		candidate b-1,3-exoglucanase		55	6	6										X	X	X			X		
108776		candidate b-1,3-glucanase; similar to *T. harzianum* BGN13.1 (GenBank acc.no.CAA58889) and *T. virens* BGN2 (AAL84695)		55	6	6										X	X						
72567	cel6A;cbh2	cellobiohydrolase	cellulolytic enzyme	6	1	1	X		X									X					
73643	cel61A	endo-1,4-glucanase EGL4	cellulolytic enzyme	61	3	2			X					X				X					
120961	cel61B	endo-1,4-glucanase	cellulolytic enzyme	61	3	2			X									X	X				
76210	abf2	a-L-arabinofuranosidase (GenBand acc. No. AAP57750)	hemicellulolytic enzyme	62	1	1	X		X	X			X						X				
22072		candidate a-glucosidase		63	2	2												X					
75036		candidate a-glucosidase		63	2	2									X								
65137		candidate b-glycosidase distantly related to b-1,3-glucanase		64	3	3									X	X	X	X	X	X	X	X	X
123639		candidate b-glycosidase distantly related to b-1,3-glucanase		64	3	3			X		X								X				
124175		candidate b-glycosidase distantly related to b-1,3-glucanase		64	3	3					X	X				X					X		
123456		candidate acid trehalase		65	2	1												X					
72526	glr1	a-glucuronidase (GenBand acc.no. CAA92949)	hemicellulolytic enzyme	67	1	1	X		X	X								X				X	
122081	cel7B	endo-1,4-glucanase EGL1; C-terminal CBM1	cellulolytic enzyme	7	2	2			X									X	X				

* total number of genes belonging to this GH family in *T. reesei*

** number of genes of this GH family being potentially regulated by light, as described in this study

GENE_ID	Gene name	Description	Specification	Glycoside Hydrolase Family	all*	here**	upregulation							downregulation							PhLP1-GNB1-GNG1 target	ENV1-BLR1-BLR2 target	shared target
							WT	Δ*phlp1*	Δ*gnb1*	Δ*gng1*	Δ*env1*	Δ*blr1*	Δ*blr2*	WT	Δ*phlp1*	Δ*gnb1*	Δ*gng1*	Δ*env1*	Δ*blr1*	Δ*blr2*			
123989	cel7A; cbh1	cellobiohydrolase		7	2	2	X	X	X	X								X					
71532		candidate a-glycosidase distantly related to a-1,3-glucanase		71	4	3			X														
108672		candidate a-1,3-glucanase		71	4	3										X		X	X	X	X		
120873		candidate a-1,3-glucanase		71	4	3												X	X				
22914		candidate b-1,3-glucanosyltransglycosylase		72	5	5												X					
77942		candidate b-1,3-glucanosyltransglycosylase		72	5	5												X					
78713		candidate b-1,3-glucanosyltransglycosylase		72	5	5	X		X										X				
82633		candidate b-1,3-glucanosyltransglycosylase		72	5	5												X					
123538		candidate b-1,3-glucanosyltransglycosylase		72	5	5												X					
49081	cel74A	candidate xyloglucanase; EGL6	hemicellulolytic enzyme	74	1	1	X		X									X					
42152		candidate b-glycosidase related to chitosanase		75	3	3									X	X					X		
66789		candidate b-glycosidase related to chitosanase		75	3	3									X	X		X		X	X	X	X
70341		candidate b-glycosidase related to chitosanase		75	3	3										X	X			X	X		
27395		candidate a-glycosidase		76	7	2					X												
55802		candidate a-glycosidase		76	7	2					X												
58887		candidate a-glycosidase distantly related to a-L-rhamnosidase		78	1	1												X	X			X	
71394		candidate b-glycosidase related to b-glucuronidase		79	4	3									X	X	X	X	X	X	X	X	X
72568		candidate b-glycosidase distantly related to b-glucuronidase		79	4	3													X				
106575		candidate b-glucuronidase		79	4	3													X				
73256		candidate b-glycosidase related to endo-b-1,3-glucanase		81	2	2										X		X	X	X		X	
79602		candidate b-glycosidase related to endo-b-1,3-glucanase		81	2	2									X	X	X						
58117		candidate a-glycosidase related to a-N-acetylglucosaminidase		89	2	2												X					
69700		candidate a-glycosidase related to a-N-acetylglucosaminidase		89	2	2												X					
57098		candidate a-glycosidase distantly related to a-1,2-mannosidase		92	7	6												X					
60635		candidate a-glycosidase distantly related to a-1,2-mannosidase		92	7	6									X	X	X	X	X				
69493		candidate a-glycosidase distantly related to a-1,2-mannosidase		92	7	6										X							
74198		candidate a-glycosidase		92	7	6									X			X					
79921		candidate a-glycosidase distantly related to a-1,2-mannosidase		92	7	6									X	X		X					
111733		candidate a-glycosidase distantly related to a-1,2-mannosidase		92	7	6									X				X				
5807		candidate a-glycosidase distantly related to a-1,2-L-fucosidase	hemicellulolytic enzyme	95	4	2											X		X				
58802		candidate a-glycosidase distantly related to a-1,2-L-fucosidase	hemicellulolytic enzyme	95	4	2										X		X		X		X	

* total number of genes belonging to this GH family in *T. reesei*

** number of genes of this GH family being potentially regulated by light, as described in this study

Supplementary Table S2 List of genes coregulated with *cel6a/cbh2*.

TRE	Gene name	Description	Specification	Group	Topic
TR_72567	cel6A;cbh2	cellobiohydrolase CBH2	cellulolytic enzyme	Glycoside Hydrolase Family 6 protein	Glycoside hydrolases
TR_102743					
TR_103041					
TR_103064		Uncharacterized conserved protein			
TR_103555					
TR_105275					
TR_108477		candidate oligo-1,6-glucosdiase		Glycoside Hydrolase Family 13 protein	Glycoside hydrolases
TR_112328					
TR_120120					
TR_120229		candidate xylanase	hemicellulolytic enzyme	Glycoside Hydrolase Family 10 protein	Glycoside hydrolases
TR_120311		unique protein			
TR_120312	cel5A	endo-1,4-glucanase EGL2	cellulolytic enzyme	Glycoside Hydrolase Family 5 protein	Glycoside hydrolases
TR_120504					
TR_120877					
TR_120961	cel61B	endo-1,4-glucanase; CEL61B	cellulolytic enzyme	Glycoside Hydrolase Family 61 protein	Glycoside hydrolases
TR_121127	bxl1	b-xylosidase BXL1 (GenBank acc. No.CAA93248)		Glycoside Hydrolase Family 3 protein	Glycoside hydrolases
TR_121189					
TR_121418					
TR_122208	xyr1	xylanase regulatator 1		cellulase transcription factor	Transcription factors
TR_123473		MFS, 12 TMs			
TR_123940		CIP2			
TR_123992		swollenin			
TR_23146					
TR_3739		candidate b-xylosidase	hemicellulolytic enzyme	Glycoside Hydrolase Family 43 protein	Glycoside hydrolases
TR_49081		candidate xyloglucanase; EGL6 (CEL74A)	hemicellulolytic enzyme	Glycoside Hydrolase Family 74 protein	Glycoside hydrolases
TR_52438					
TR_53238		TRE456, hypothetical G-protein coupled receptor, family 2, secretin like, related to Magnaporthe grisea MG00532 (GPCR with homology to rat growth hormone releasing factor)		G-proteins	signal trans-duction
TR_53331					
TR_53722					
TR_54086					
TR_54219					

TRE	Gene name	Description	Specification	Group	Topic
TR_55999		candidate a-galactosidase		Glycoside Hydrolase Family 27 protein	Glycoside hydrolases
TR_56684		hypothetical sugar transporter family protein			Transport
TR_61763					
TR_63001					
TR_69276		candidate b-glycosidase distantly related to xylanase		Glycoside Hydrolase Family 5 protein	Glycoside hydrolases
TR_69316		unknown conserved protein			
TR_69375		unknown hypothetical protein			
TR_69944		candidate a-xylosidase		Glycoside Hydrolase Family 31 protein	Glycoside hydrolases
TR_70098		Amino acid transporters			
TR_70520					
TR_72526		candidate a-glucuronidase GLR1 (GenBand acc.no. CAA92949)	hemicellulolytic enzyme	Glycoside Hydrolase Family 67 protein	Glycoside hydrolases
TR_73632		AXE1			
TR_73638		CIP1			
TR_73654		The basic-leucine zipper (bZIP) transcription factors are proteins that contain a basic region mediating sequence-specific DNA-binding followed by a leucine zipper region (see IPR002158) required for dimerization.	basic-leucine zipper domain-containing/DNA binding domain protein		Transcription factors
TR_76215		Sulfide dehydrogenase		Additional aspects of sulfur metabolism	Sulfur meta-bolism
TR_76696		Flavin-containing monooxygenase			
TR_76722		flavohemoglobin			
TR_77154		RfeB is an A. nidulans DNA-binding Homeobox Nuclear protein	transcription factor-like protein		Trans-cription factors
TR_77481		D-xylulose 5-phosphate/D-fructose 6-phosphate phosphoketolase			
TR_80240	bga1	b-galactosidase (GenBank acc. No. CAD70669) (EC 3.2.1.23)		Glycoside Hydrolase Family 35 protein	Glycoside hydrolases
TR_81148					

Supplementary Table S3 - Sequences of oligonucleotides used in this study.

fragment	oligonucleotide	study	sequence
cbh1	RTcbh1F	Tisch *et al*., 2011a	5' ACCGTTGTCACCCAGTTCG 3'
	RTcbh1R	Tisch *et al*., 2011a	5' ATCGTTGAGCTCGTTGCCAG 3'
rpl6e	RTL6eF1	Tisch *et al*., 2011a	5' GATACGTCATCGCCACCTCC 3'
	RTL6eR1	Tisch *et al*., 2011a	5' CTTCTCCTTGGCCTTCTCG 3'
xyl1	RT_xyl1_fw1	This study	5' CATCTACGCCCGCATCAAGC 3'
	RT_xyl1_rev1	This study	5’ CAAAGGACGAGTAGGCGGTGAC 3’
lad1	RT_lad1_fw1	This study	5’ CTTGCCCGCTTGTCATCAC 3’
	RT_lad1_rev1	This study	5’ CCGTTTCCTCAGCCGACTTG 3’
xdh1	RT_xdh1_fw1	This study	5’ CTCCACCATTGTCAGCGTC 3’
	RT_xdh1_rev1	This study	5’ CCTTTGCGTTGTCCTCAGC 3’
lxr3	RT_lxr3_fw1	This study	5’ AGGAGGGCACGACGATTGAC 3’
	RT_lxr3_rev1	This study	5’ GCCTTGGAGCTGTTGTACACG 3’
lxr4	RT_lxr4_fw1	This study	5’ GGCAAGAAGTTCCAGGTCG 3’
	RT_lxr4_rev1	This study	5’ CAGCACGTTGATGGCGTAG 3’
xki1	RT_xki1_fw1	This study	5’ TGTCAGATCACGCCCTTCAC 3’
	RT_xki1_rev1	This study	5’ GGTTGAAGAAGTGGTACGAGC 3’
